# Comparative Structural Dynamics of Isoforms of *Helicobacter pylori* Adhesin BabA Bound to Lewis b Hexasaccharide *via* Multiple Replica Molecular Dynamics Simulations

**DOI:** 10.3389/fmolb.2022.852895

**Published:** 2022-05-02

**Authors:** Rajarshi Roy, Nisha Amarnath Jonniya, Md Fulbabu Sk, Parimal Kar

**Affiliations:** Department of Biosciences and Biomedical Engineering, Indian Institute of Technology Indore, Indore, India

**Keywords:** conformational dynamics, multiple replica molecular dynamics simulations, BabA, free energy landscape, principal component analysis, phi/psi maps, loop dynamics

## Abstract

BabA of *Helicobacter pylori* is the ABO blood group antigen-binding adhesin. Despite considerable diversity in the BabA sequence, it shows an extraordinary adaptation in attachment to mucosal layers. In the current study, multiple replica molecular dynamics simulations were conducted in a neutral aqueous solution to elucidate the conformational landscape of isoforms of BabA bound to Lewis b (Le^b^) hexasaccharide. In addition, we also investigated the underlying molecular mechanism of the BabA-glycan complexation using the MM/GBSA scheme. The conformational dynamics of Le^b^ in the free and protein-bound states were also studied. The carbohydrate-binding site across the four isoforms was examined, and the conformational variability of several vital loops was observed. The cysteine–cysteine loops and the two diversity loops (DL1 and DL2) were identified to play an essential role in recognizing the glycan molecule. The flexible crown region of BabA was stabilized after association with Le^b^. The outward movement of the DL2 loop vanished upon ligand binding for the Spanish specialist strain (S381). Our study revealed that the S831 strain shows a stronger affinity to Le^b^ than other strains due to an increased favorable intermolecular electrostatic contribution. Furthermore, we showed that the α1-2-linked fucose contributed most to the binding by forming several hydrogen bonds with key amino acids. Finally, we studied the effect of the acidic environment on the BabA-glycan complexation *via* constant pH MD simulations, which showed a reduction in the binding free energy in the acidic environment. Overall, our study provides a detailed understanding of the molecular mechanism of Le^b^ recognition by four isoforms of *H. pylori* that may help the development of therapeutics targeted at inhibiting *H*. *pylori* adherence to the gastric mucosa.

## Introduction

The mucus layer of the stomach in humans represents the first barrier for gastrointestinal bacteria. It consists of mucin MUC5AC and MUC6, secreted from the surface mucosa and gland mucosa, respectively (Ho et al., 1995). These secreting mucins are heavily O-glycosylated glycoproteins (Strous and Dekker, 1992; Nordman et al., 1995). One of the most common gastric pathogens colonizing the stomach mucosa is *Helicobacter pylori*. It affects more than half of the global population (Cave, 1996). It is a Gram-negative, microaerophilic, spiral bacteria that has been identified as a major etiological factor in gastritis, gastric and duodenal ulcers, gastric adenocarcinoma, and gastric-mucosa-associated lymphoid tissue (MALT) lymphoma (Schreiber et al., 2004; Polk and Peek, 2010). It can thrive in harsh conditions of the stomach by the adaptive mechanism of using a variety of outer membrane proteins to adhere with glycan moieties on the gastric epithelium. This mechanism helps *H*. *pylori* to sustain local colonization and adherence onto gastric epithelium (Ilver, 1998).

Adhesin of *H*. *pylori* to mucin and host cells is highly relevant for the progression of the disease, colonization, and mucin-mediated response (Sheu, 2003; Yamaoka, 2008; Ishijima et al., 2011). Several molecules have been implicated as receptors for H. *pylori* adhesins (Evans and Evans, 2000). *H*. *pylori* adheres to the glycans present at high density in glycolipids, glycoproteins, and mucins of the gastrointestinal tract (GI). Primarily, it interacts *via* the blood group antigen-binding adhesin (BabA), which binds to mono (ABO) and di-fucosylated (Lewis b; Le^b^) derivatives of type 1 O-glycan structures. On the other hand, the sialic acid-binding adhesin (SabA) binds to sialyl-Lewis x (SLe^x^) and sialyl-Lewis a (SLe^a^) (Lindén et al., 2002, 2004). The most studied interaction is between BabA and Le^b^ (Ilver, 1998). This di-fucosylated oligosaccharide is abundantly expressed in gastric mucosa, including the O phenotype population. They are most susceptible to peptic ulcer disease (Boren et al., 1993; Lindén et al., 2002). BabA belongs to the *Helicobacter* outer membrane porins (Hop), adhering to glycan moieties on the gastric epithelium (Alm et al., 2000; Hage et al., 2015).

The *H*. *pylori* strain variants determine the longevity of the established infection and disease outcomes (Kang and Blaser, 2006; Suerbaum and Josenhans, 2007). Furthermore, the increased genetic diversity of *H*. *pylori*, particularly the high adaptive evolution in the BabA adhesin, affects binding preference to the blood group strains such as the ABO versus O, depicting as “generalist” versus “specialist” preference, respectively (Kang and Blaser, 2006). It is also evident that BabA’s adaptive traits, such as the specialist strains, are more likely to bind to Le^b^ and blood group O dominant populations. In contrast, the generalist strains can bind Le^b^ and the GalNAc- and Gal-forms of blood group A (ALe^b^) and B (BLe^b^) derivatives. In particular, *H*. *pylori* strains have displayed a 1,000-fold range of affinities against Le^b^ glycan receptors (K_a_ ∼10^8^ to 10^11^ M^−1^) (Aspholm-Hurtig, 2004).

**FIGURE 1:**
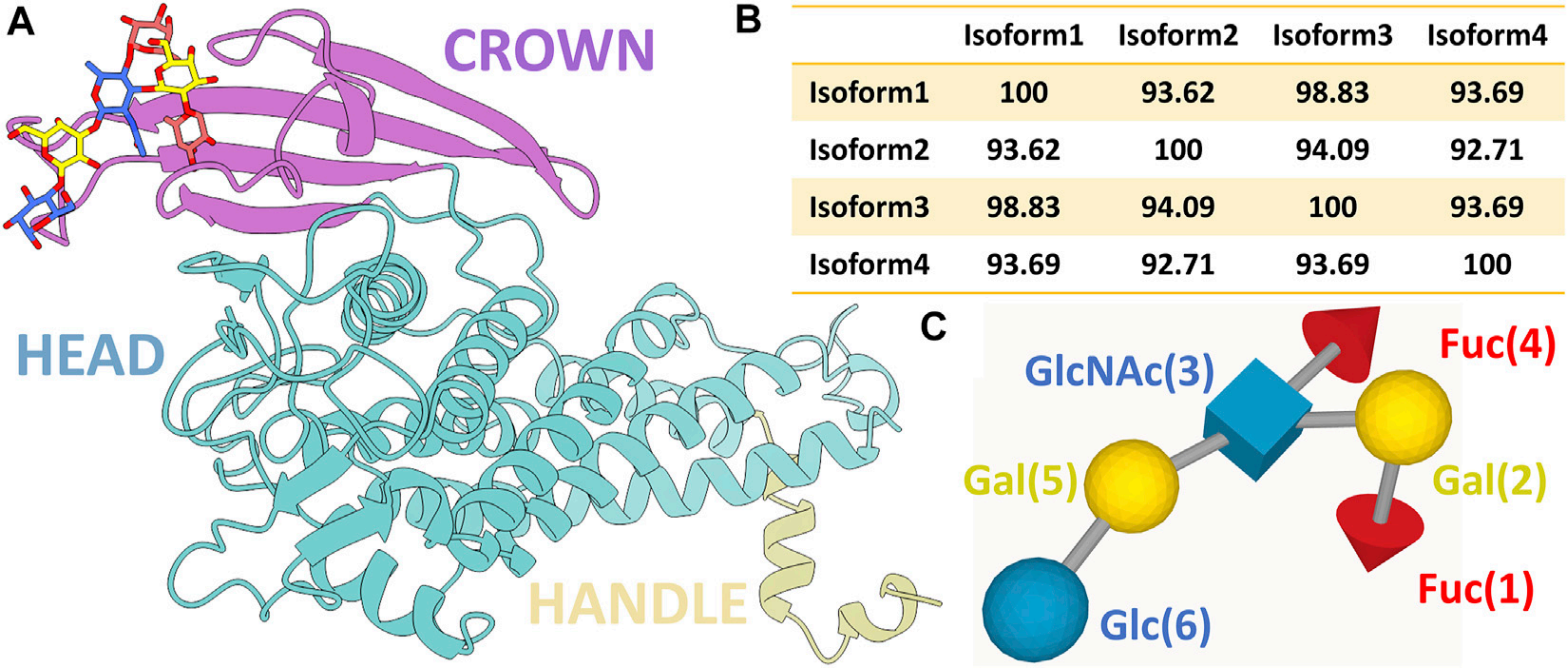
**(A)** Ribbon representation of BabA protein with the bound Le^b^ (ball and stick representation). Three different regions of the protein, namely, crown, head, and handle, are shown in distinct colors. **(B)** Percentage probability of the sequence alignment between all four isoforms. **(C)** SNFG representation of the Lewis sugar b (Le^b^).

Structurally, BabA contains an N-terminal extracellular host-binding domain and a C-terminal outer membrane domain, forming a *β*-barrel structure (Alm et al., 2000). As depicted in Figure 1A, BabA also contains two *α*-helical regions-a handle and a head region. On top of the head region, there is a *β*-sheet known as the crown. The handle region containing the *α* and *β* unit of the extracellular domain, the highly conserved β-barrel transmembrane domain, succeeds the *α*-C2 helix of the handle region. The core of the head region is primarily comprised of four helices, which are at a perpendicular angle to the handle region, making a markedly kinked tertiary structure. The crown is composed of four antiparallel *β*-sheets at the highest tip of the protein, which consists of the important Le^b^ binding sites (Doohan et al., 2021).

In contrast to BabA, there is a complete absence of a four-strand antiparallel β-sheet of the crown region in SabA, which recognizes sialyl-Lewis^x^ (SLe^x^) found on the cancerous and infected gastric tissues (Pang et al., 2014). The multiple sequence analysis of BabA isoforms shows ∼93% sequence diversity (Figure 1B and Supplementary Figure S1). It has been observed that Le^b^ interacts with the crown of BabA (Hage et al., 2015). Le^b^ is comprised of two fucose residues (Fuc1 and Fuc4), two galactose residues (Gal2 and Gal5), an *N*-acetyl glucosamine residue (GlcNAc3), and a glucose residue (Glc6) (see Figure 1C). However, each isoform’s structure to molecular determinants and specificity toward Le^b^ is unknown.

This study focuses on understanding the BabA adhesin sequence diversity among different strains (strain 17875; Isoform 1, strain P436; Isoform 2, strain S831; Isoform 3, and strain A730; Isoform 4) toward Le^b^-type glycan receptor binding and elucidating the biophysical basis of their molecular recognition and conformational dynamics. The atomistic description provided in the study helps to understand structural and binding differences in BabA’s carbohydrate-binding domain (CBD) for each isoform. We investigated the conformational dynamics of various functional loops, such as Cysteine bound loops (CL) and Diversification loops (DL), which exhibit high polymorphism in *H*. *pylori* isolates toward Le^b^ binding strength. In addition, we studied the conformational dynamics of Le^b^ and elucidated the glycan-binding mechanism *via* molecular mechanics generalized Born surface area (MM/GBSA).

The *H. pylori* infection occurs in the gastric epithelium, where its mucus layer contains pH ranges from neutral to acidic. However, using chemotaxis motions, *H. pylori* moves from an acidic to a neutral environment. The binding phenomenon is acid sensitive, and the binding affinity reduces with a lowering pH value. To investigate the effect of an acidic environment on the binding, we conducted constant pH MD simulations for Iso1 as a test case. Altogether, this study provides molecular insights into the recognition and binding preference of Le^b^ across *H. pylori* BabA variants.

## Materials and Methods

### MD Simulations of BabA–Le^b^ Complexes

The following four isoforms of BabA complexed with Le^b^ were used in the current study: 1) Iso1 (PDB ID 5F7M), 2) Iso2 (PDB ID 5F9A), 3) Iso3 (PDB ID 5F8R), and 4) Iso4 (PDB ID 5F93) (Moonens et al., 2016). We also simulated apo-BabA (without Le^b^) and Le^b^ in an aqueous solution. The AMBER ff14SB force field (Maier et al., 2015) was used for the protein modeling, and glycam06j-1 (Kirschner et al., 2008) was employed to describe Le^b^. The protonation state of each residue was estimated using the Propka 3.1 webserver (Olsson et al., 2011), corresponding to pH seven for all four different Isoforms of BabA. Each system was solvated in a truncated octahedral box using the TIP3P (Price and Brooks, 2004) water molecules. The distance between the wall and solute was kept as 10 Å. Each solvated system was neutralized by adding an appropriate number of counterions. All bonds, including hydrogen atoms, were restrained using the SHAKE algorithm (Kräutler et al., 2001). The long-range electrostatic interactions were treated using the particle mesh-Ewald (PME) method (Darden et al., 1993). A cut-off distance of 10 Å was set for non-bonded interactions. The temperature was maintained using the Langevin thermostat with a collision frequency of 2 ps^−1^ and the pressure using the Berendsen barostat (Berendsen et al., 1984). Two stages of minimization were performed using the steepest descent followed by the conjugate gradient algorithm for 500 steps with a harmonic restraint of 2 kcal mol^−1^ Å^−2^. It was then subjected to gradual heating from 0 to 300 K with a restraint force of 2 kcal mol^−1^ Å^−2^. Before the production run, we conducted a 1 ns simulation at the NPT ensemble without restraint on the solute for equilibration. Finally, the production simulations for complexes and apo-BabA were performed in the NPT ensemble with a timestep of 2 fs for 1 *µ*s using the *pmemd*.*cuda* module of AMBER18(Case et al., 2018). We recorded the coordinates every 10 ps, resulting in 100,000 configurations. To ensure better sampling, we conducted two more replica simulations for apo and complex systems. We also conducted 2 × 3 *µ*s long simulations for the free glycan in the aqueous solution using a similar protocol as discussed in our previous work (Roy et al., 2022).

In addition, to investigate the role of an acidic environment on the complexation of BabA/Le^b^, we conducted constant pH molecular dynamics (CpHMD) simulation at pH 4.5 for 2 × 250 ns for Iso1 by titrating acidic amino acids in the crown region (Donnini et al., 2011). In the selected region, six ionizable residues were found (E80, E158, D161, D172, D199, and D213), which we attempted to titrate during the simulation. After every 180 fs of explicit MD simulation, the protonation change event was attempted for all mentioned residues in the GB implicit model (Mongan et al., 2004).

### Analysis

All the trajectories were analyzed using the *cpptraj* module of AmberTools19 (Roe and Cheatham, 2013). The first 200 ns data from each run were discarded for the trajectory analysis to minimize initial noises. After that, the combined trajectories for three replicas were prepared and subjected to various calculations related to the dynamics of the systems. The initial stability and flexibility were estimated by calculating the root mean squared deviations (RMSD) and the root mean squared fluctuations (RMSF) concerning the well-equilibrated conformations. Conformational space of the sugar molecules in the free and bound state was also estimated with the help of glycosidic dihedral angle, Cremer–Pople (CP) (Cremer and Pople, 1975) parameters of ring puckering. The glycosidic dihedral angles for the *α*/*β*-n linkages are defined as $\varphi =O_{5}-C_{1}-O-C_{n}$ and $\psi =C_{1}-O-C_{n}-C_{\left(n-1\right)}$ . The puckering state of any sugar as per the Cremer–Pople convention was estimated using the spherical coordinate set, *Q*, *θ,* and *φ*. The spherical space was further subdivided into 38 IUPAC recognized regions, where each space is dedicated to a specific ring conformation. To estimate the global and local conformations of several loops related to binding, the Cartesian principal component analysis (PCA) and dihedral principal component analysis (dPCA) were performed, which were discussed in detail in our previous study (Roy et al., 2021). The coordinate of $C_{\alpha }$ atom of each amino acid and dihedral angle of peptide bonds of the selected loop region were used for PCA and dPCA, respectively. Furthermore, the free energy surface (FES) was constructed using the Boltzmann equation: Δ*G* = *−k*_*B*_ *T ln(ρ) - k*, where *k*_B_ is the Boltzmann constant, *T* is the temperature, and *ρ* is the probability density of the geometric coordinate *x*. The parameter (*k*) was chosen such that the global minimum was located at 0 kcal/mol. In addition, a distance cut-off of ≤3.5 Å and an angle cut-off of ≥120° were used to evaluate the hydrogen bonding (Jeffrey, 1997).

### Binding Free Energy Calculations Using the MM/GBSA Scheme

The molecular mechanics/generalized Born surface area (MM/GBSA) scheme (Onufriev et al., 2004) was employed to estimate binding free energy. For protein-glycan systems, it has been reported that MM/GBSA outperforms the computationally expensive MM/PBSA scheme (Mishra and Koča, 2018; Sood et al., 2018; Roy et al., 2020a). It offers a good trade-off between speed and accuracy compared to other computationally expensive methods, such as thermodynamics integration (TI) and free energy perturbation (FEP). The methodological details of the MM/GBSA protocol were discussed in our previous works (Kar et al., 2013; Roy et al., 2020b; Jonniya et al., 2021). The binding free energy ( $\Delta G_{binding}$ ) was determined from the free energies of the receptor/BabA (protein), the ligand (carbohydrate), and the complex (complex) according to the following equation (Kollman et al., 2000):

$$\Delta G_{binding}=\;G_{complex}-\left(G_{carbohydrate}+\;G_{protein}\right)$$ (1)

The free energy of each species (protein, carbohydrate, and complex) was estimated using the following formula:

$$G=E_{vdW}+E_{ele}+G_{GB}+G_{SA}$$ (2)

where $E_{vdW}$ and $E_{ele}$ represent the van der Waals and electrostatic interaction energy, respectively. The polar solvation free energy ( $G_{GB}$ ) was estimated using the generalized Born equation, while the non-polar solvation free energy $\left(G_{SA}\right)\;$ was determined using the following equation:

$$G_{SA}=\;\gamma A+b$$ (3)

with $\gamma =0.00542\;\text{kcal}.{\text{mol}}^{-1}.{\text{Å}}^{-2}$ and $b=0.92\text{ kcal}.{\text{mol}}^{-1}$ . The symbol *A* denotes the solvent-accessible surface area (SASA). The GB model (*igb* = 2) developed by Onufriev et al. was used in this study (Onufriev et al., 2004). The configurational entropy was not considered in the current study due to the high computational cost. We considered 25000 frames from the last 500 ns of each production simulation for the free energy calculation. The overall binding free energy was estimated by averaging the binding free energies obtained from the three independent runs. In addition, we performed a per-residue decomposition of the binding free energy by the MM/GBSA scheme. On the other hand, 7500 frames from the last 150 ns of the trajectory were employed for the binding free energy calculation at pH 4.5. The final values were determined by averaging both replica runs.

## Results and Discussions

### Structural Stability and Flexibility Analyses in Neutral Aqueous Solution

**FIGURE 2:**
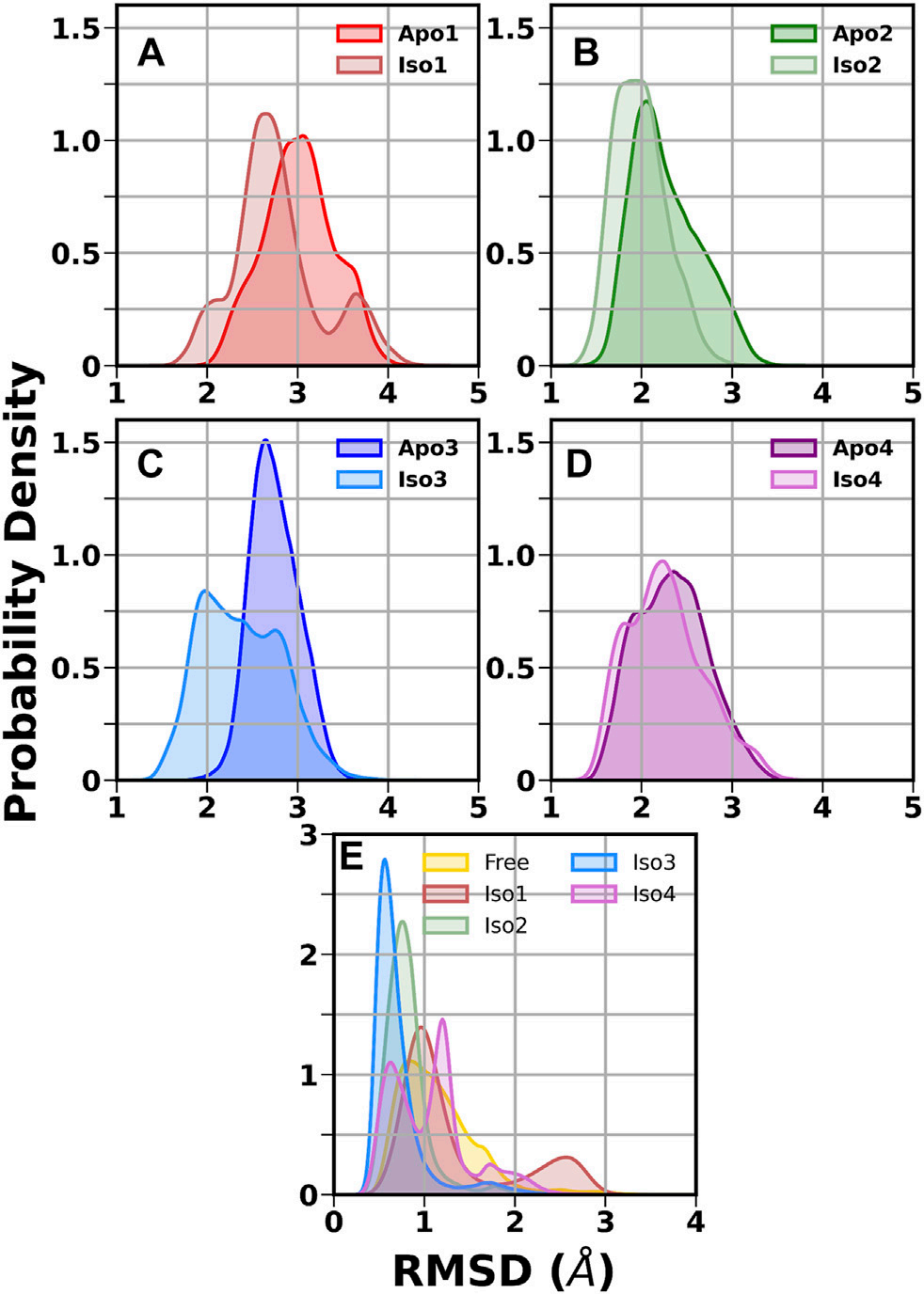
Distribution of root mean squared deviation (RMSD) of the crown and head region. **(A)** Iso1 (Apo and Complex), **(B)** Iso2 (Apo and Complex), **(C)** Iso3 (Apo and Complex), **(D)** Iso4 (Apo and Complex), and **(E)** free and bound Le^b^.

First, we investigated the structural stability of each system by analyzing the root mean squared deviations (RMSD) with respect to the corresponding crystal structure. The time evolution of RMSD for multiple runs is presented in Supplementary Figures S2-S3. It demonstrates that simulations are stable for each BabA isoform in its apo (see Supplementary Figure S2) and glycan-bound forms (Supplementary Figure S3). The handle region of BabA was more flexible as it was not fully crystallized, which led to an increase in the overall RMSD. Next, we determined the RMSD of each BabA–Le^b^ complex isoform by excluding the handle region and computed the corresponding probability distribution by combining all three replica runs (see Figure 2). In addition, each complex was compared with the respective free form. The average RMSD values for multiple runs were computed and listed in Table 1. It is evident from Table 1 that the average value varies between 2.96 Å and 3.60 Å for all apo isoforms, while the RMSD value decreases upon complexation, with the values ranging from 2.27 to 3.16 Å for all four complexes. It is evident from Figure 2A-D and Table 1 that compared to apo, BabA–Le^b^ complexes got relatively stabilized. However, for Iso4, a slightly higher RMSD was observed. These results indicate that the interaction between Le^b^ and BabA leads to overall structural stability. All complexes except Iso3 showed similar distribution for apo and bound states. In Iso3, the apo form showed a stable conformation across all three independent runs, which got relatively flexible after the binding of Le^b^. In the apo case, the distribution was narrow with a sharp peak, while a broader distribution was observed when a complex was formed. However, the peak shifted to a lower RMSD value compared to apo Iso3.

**TABLE 1:** The average root mean squared deviation (RMSD), radius of gyration (RoG), and solvent accessible surface area (SASA) over three replica runs using the block average method. The standard error of the mean is given in parentheses.

RMSD (Å)		RoG (Å)		SASA (nm^2^)	
Apo	Complex	Apo	Complex	Apo	Complex
3.60 (0.03)	3.09 (0.04)	26.68 (0.02)	26.37 (0.02)	203.47 (0.28)	196.11 (0.27)
2.96 (0.05)	2.27 (0.02)	26.36 (0.03)	26.59 (0.01)	200.93 (0.33)	197.11 (0.23)
3.20 (0.02)	2.96 (0.04)	26.42 (0.02)	26.43 (0.02)	201.50 (0.33)	200.63 (0.28)
3.07 (0.02)	3.16 (0.05)	26.88 (0.02)	26.73 (0.04)	208.69 (0.43)	202.56 (0.27)

Next, we calculated the RMSD of the ring atoms of Le^b^ in the free and protein-bound states concerning the initial coordinates. The corresponding RMSD distribution is displayed in Figure 2E. A broad distribution was observed for the free glycan with a mean value of ∼0.9 Å. In the case of the Iso3 complex, a very sharp unimodal distribution with a peak at ∼0.5 Å was observed, indicating a firm binding. A similar distribution was observed for Iso2/Le^b^. On the other hand, moderate flexibility was observed for the glycan when bound to Iso1 and Iso4. In the case of Iso1, the most probable peak is located at 1.0 Å, with a secondary peak at ∼2.6 Å. In the case of Iso4, the RMSD distribution is characterized by two peaks, situated at ∼0.5 Å and ∼1.1 Å. The degree of flexibility of the glycan in the protein-bound form may reflect its binding affinity, as discussed in the later section.

Next, the average values of the radius of gyration (RoG) were computed to check the compactness of the protein and are listed in Table 1. The time evolution of RoG of each isoform in free and ligand-bound states is shown in Supplementary Figures S4, S5. It is evident from Supplementary Figure S5 that in the case of Iso4, run2 showed distinct time series compared to other runs. It is worth mentioning that the deviation in RoG was mostly because of the flexible handle region, which is located far from the binding region. Overall, the calculated average RoG values indicated no significant changes for each isoform. Next, we computed the SASA and found that the SASA value of apo variants was more significant than their respective complex form, as seen in Table 1 and Supplementary Figures S6, S7. The distribution of RoG and SASA for apo and complexes are shown in Supplementary Figure S8. These observations agree with the RMSD profile, suggesting that non-polar amino acid residues form interactions during complexation with the glycan. In addition, it suggests that the overall stability of BabA is enhanced upon complexation with the glycan, indicating that the hydrophobic core of BabA is more protected from the external environment upon binding with Le^b^ (see Supplementary Figure S8).

**FIGURE 3:**
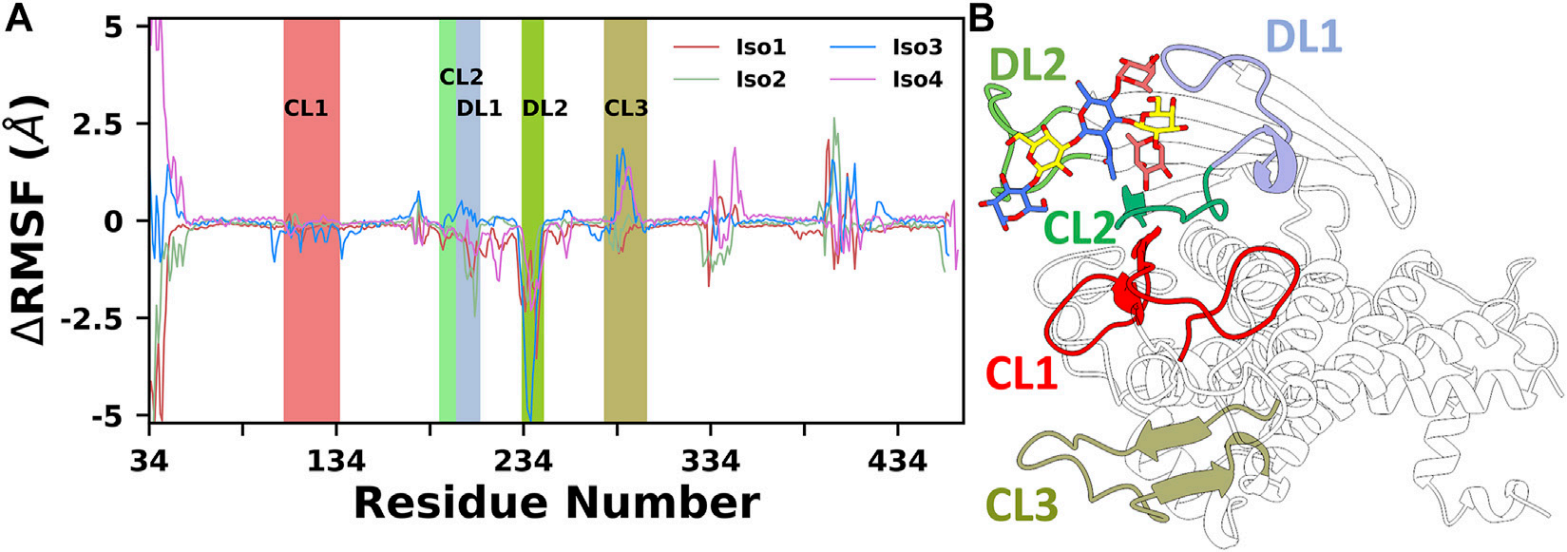
**(A)** Difference in root mean squared fluctuation (RMSF) of four isoforms with their respective Apo conformation. Different loop regions are highlighted in the plot. **(B)** Location of all key loop regions in BabA.

Finally, to provide further insights into the flexibility of different regions of BabA, the root means square fluctuations (RMSF) of C_α_ atoms were computed. The difference RMSF (∆RMSF) plot of the complex from the respective apo conformation was computed and shown in Figure 3. The N-terminal domain of BabA was relatively more flexible, and it corresponds to the handle region. However, this region is located far from the binding site and can be ignored for our current study. Furthermore, it is evident from Figure 3 that among different significant loop regions of BabA, DL2 of apo Iso3 exhibited large fluctuations compared to the corresponding complex. This indicates that the DL2 loop underwent structural changes upon interaction with Le^b^ and could play a crucial role in the molecular specificity of Le^b^ across isoforms of the *H*. *pylori* adhesin BabA.

Overall, Iso3 showed differences in dynamics and flexibility upon complexation, especially near the binding site, compared to the other three isoforms. This structural change may influence the Le^b^ recognition process.

### Conformational Dynamics of Free and Protein-Bound Le^b^ in Neutral Aqueous Solution

**FIGURE 4:**
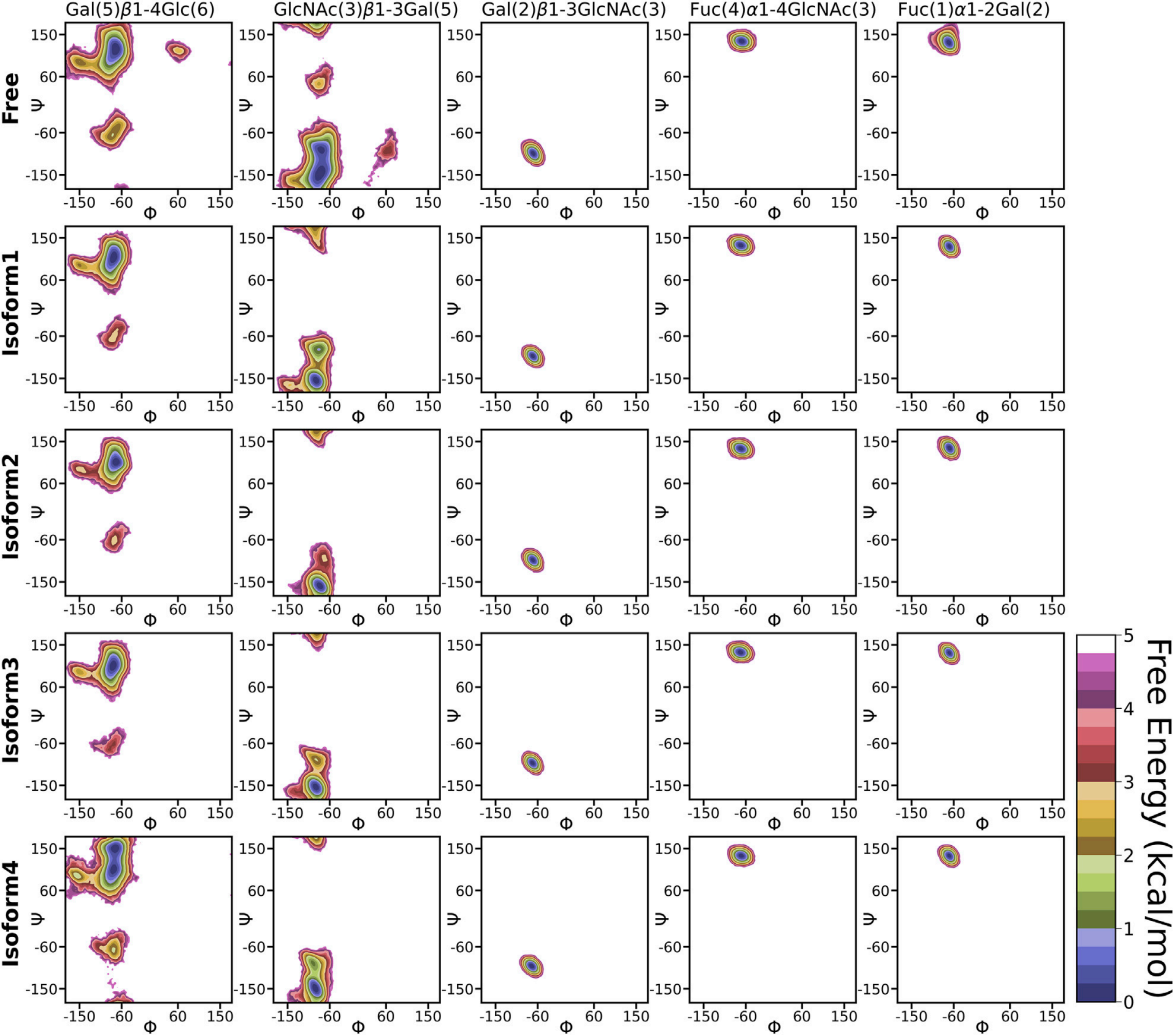
2D free energy surface constructed using dihedral angles φ/ψ of glycosidic angle in the free and bound form of Le^b^.

The flexible glycosidic linkage of Le^b^ yields several conformations, which may play a critical role in protein-glycan recognition. The flexibility was investigated by calculating the free energy surface (FES) using *φ*/*ψ* angles of each glycosidic linkage and shown in Figure 4. The flexibility of each linkage stays similar for free and bound states. Although the conformational sampling across the dihedral space remained almost unchanged after Le^b^ binding, a minor variation for specific linkages, such as [Gal (5)*β*1-4Glc (6)] and [GlcNAc(3)*β*1-3Gal (5)], was observed. Both linkages displayed higher flexibility compared to others. Furthermore, linkages such as Fuc (4)*α*1-4GlcNAc(3), Fuc (1)*α*1-2Gal (2), and Gal (2)*β*1-3GlcNAc(3) stayed at a single minimum along with a very narrow conformational basin irrespective of free or bound states. The GlcNAc(3)*β*1-3Gal (5) linkage showed two equiprobable minima around (−80, −150) and (−80, −80), which got diminished after ligand binding. In the bound state, the flexibility of Le^b^ remained almost similar across all isoforms, indicating a stable conformation around the binding pocket. However, in the case of Iso4, the [Gal (5)*β*1-4Glc (6)] linkage showed two closely separated minima regions, indicating a slightly different conformation.

**FIGURE 5:**
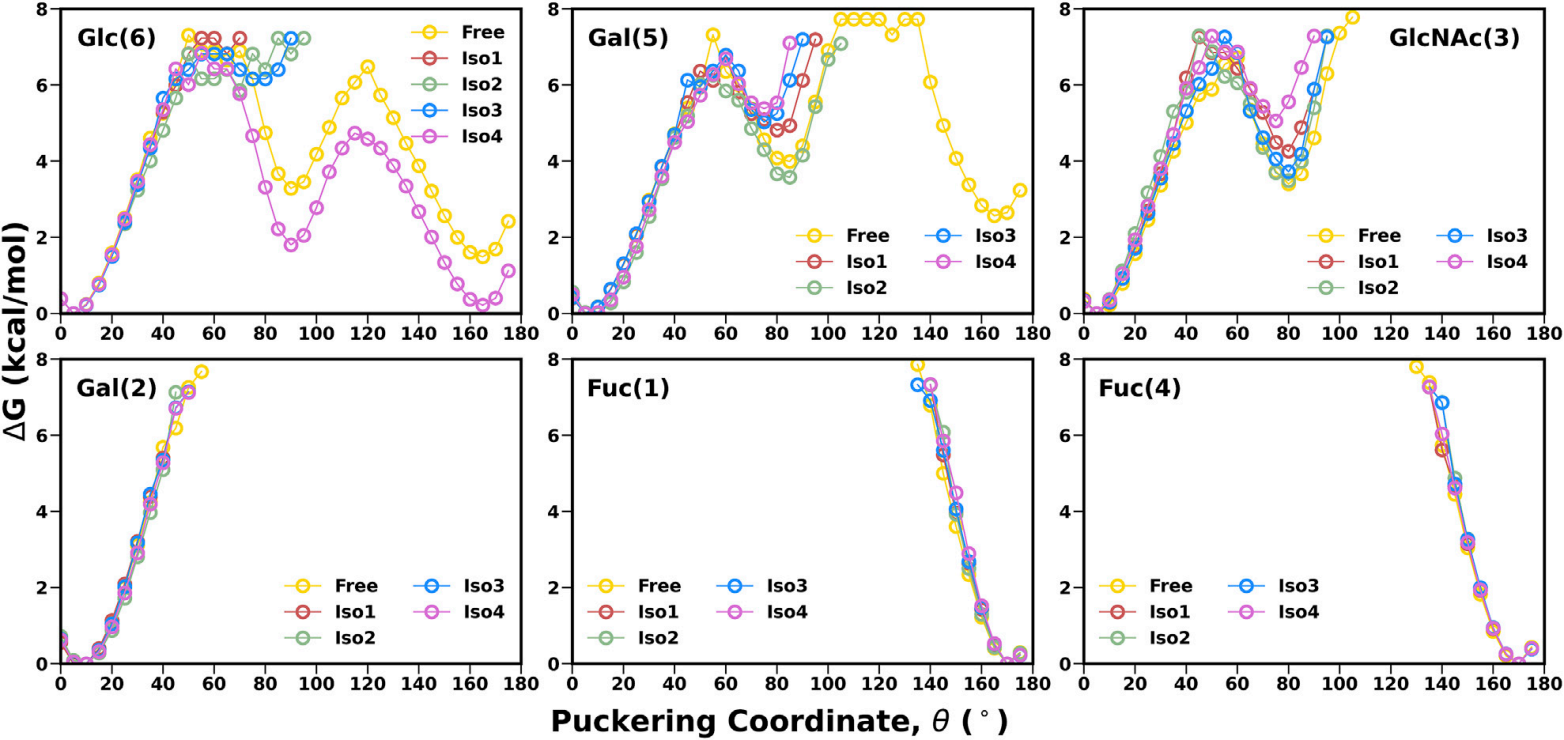
1-D free energy surface generated using Cremer–Pople θ puckering profiles of each monosaccharide of Le^b^.

Another important parameter for characterizing the carbohydrate conformation is the ring geometry, for which we evaluated the puckering profile for each monosaccharide. We used the Cremer–Pople parameter (*θ*) to construct the conformational free energy to show the transition from ^4^C_1_ to ^1^C_4_. In our study, no monosaccharide was able to complete the chair (^4^C_1_)-to-chair (^1^C_4_) transition, except for glucose (6) and galactose (5) in the free system (see Figure 5). However, other galactose, such as Gal (2), stayed around the ^4^C_1_ conformer, indicating a similar rigidity as other studies (Alibay et al., 2018). In the case of *β*-glucose, the complete transition was observed in the free simulation where the ^1^C_4_ state is energetically much higher than the other chair form ^4^C_1_ (∼2 kcal/mol). On the contrary, in the case of Iso4, both the pucker states were sampled in an equiprobable way. The flexible nature of this ring conformation was also reflected in the dihedral space, as shown in the FES of the [Gal (5)*β*1-4Glc (6)] linkage (Figure 4). An earlier study also reported a change in the puckering state for glucose in a microsecond timescale (Alibay and Bryce, 2019). Fucose residues stayed only in the ^1^C_4_ state, as observed in another protein–carbohydrate recognition study (Roy et al., 2020a). Overall, Le^b^ displayed similar flexibility across all four isoforms.

### Conformational Flexibility of the Crown Region

**FIGURE 6:**
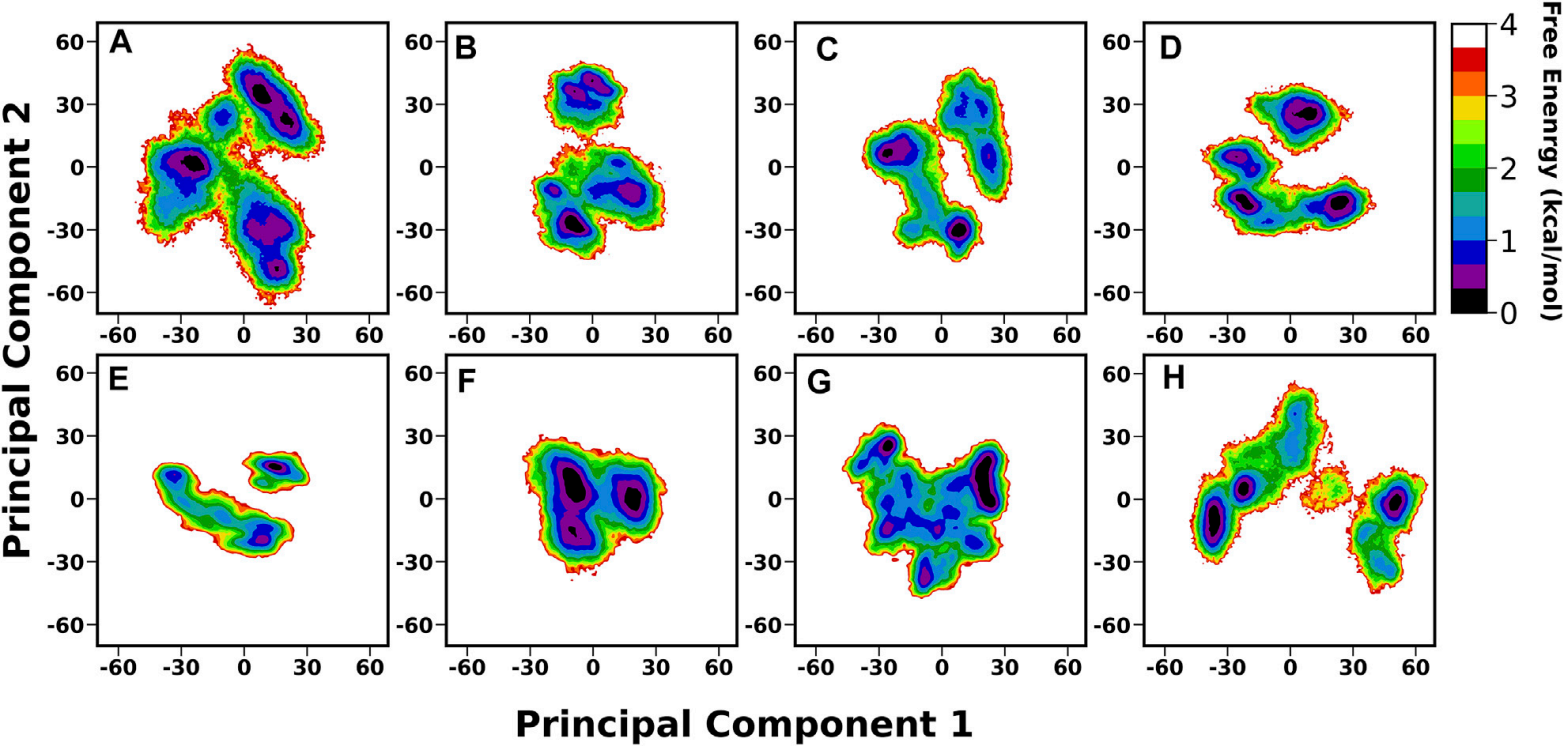
Free energy analysis of complete BabA with respect to Cα principal components, where PC1 and PC2 represent the first and second principal components of motion. The first row represents the Apo cases, **(A)** Iso1, **(B)** Iso2, **(C)** Iso3, and **(D)** Iso4, whereas the second row represents the complex simulations, **(E)** Iso1, **(F)** Iso2, **(G)** Iso3, and **(H)** Iso4.

A thorough structural investigation was conducted to gain insights into the dynamicity of the crown region of BabA, which mainly interacts with the glycan molecule. This region is comprised of several key loops, such as CL1-3 and DL1-2, which are the key functional regions for the glycan binding. The principal component analysis (PCA) of BabA was conducted, and the free energy landscape was generated using the first two principal components (see Figure 6). The apo structures of all four BabA isoforms showed several co-existing minima connected *via* shallow energy barriers, suggesting a very dynamical system in solution. However, upon Le^b^ binding, these regions shrunk to a certain extent, indicating the stability of the protein–carbohydrate complexes, especially for Iso1 and Iso2. In addition, for Iso4, both in apo and bound states, several minima regions were separated by a high energy barrier (more than 4 kcal/mol). However, the Cartesian coordinate-based PCA fails to provide a correct separation of internal and overall motions. The projection on the low-dimensional space offers only limited information about the multidimensional energy landscape (Krivov and Karplus, 2004).

**FIGURE 7:**
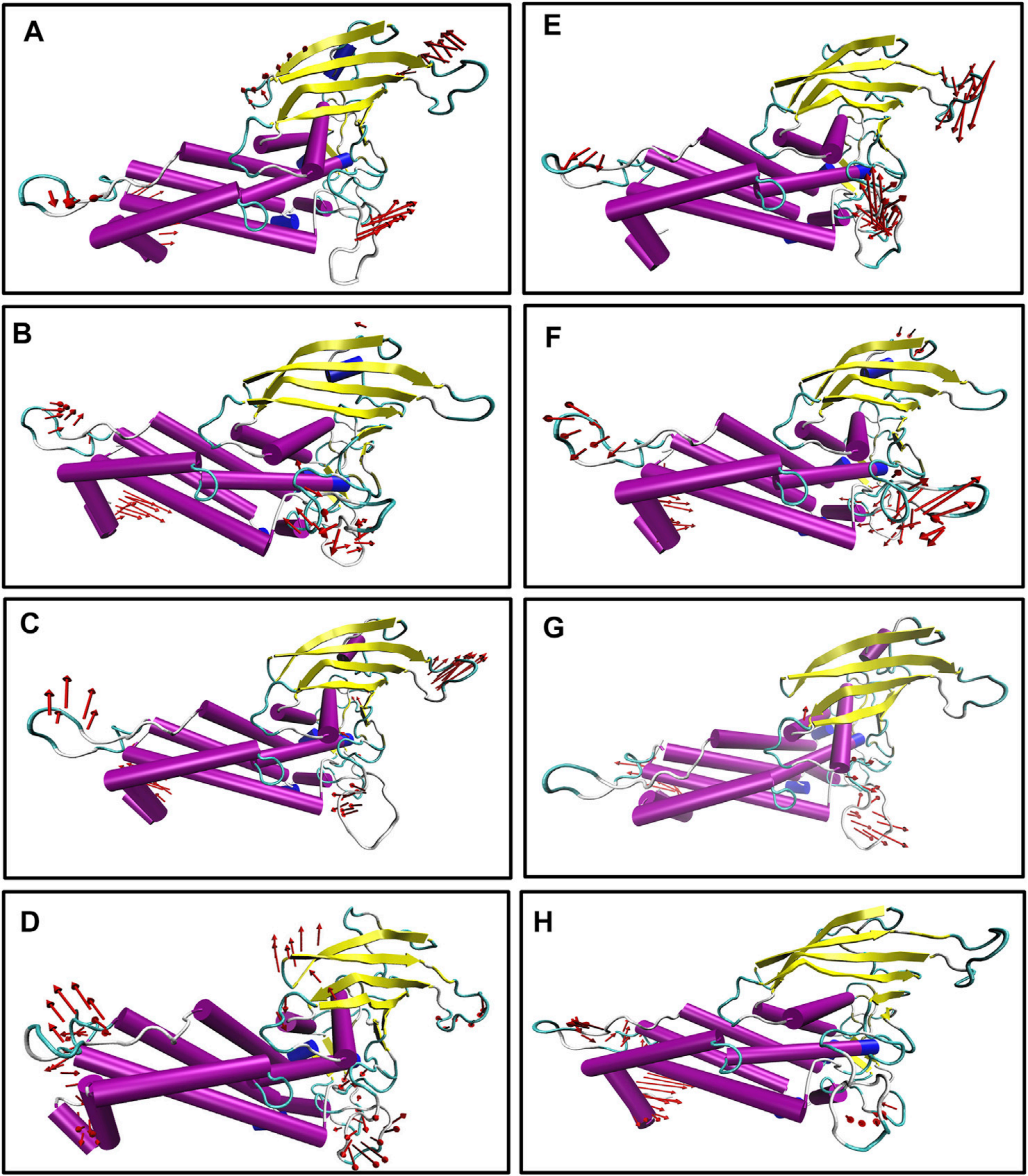
Porcupine plot corresponding to PC1 obtained by performing principal component analysis on MD trajectories, **(A)** Iso1 (Apo), **(B)** Iso2 (Apo), **(C)** Iso3 (Apo), **(D)** Iso4 (Apo), **(E)** Iso1 (Complex), **(F)** Iso2 (Complex), **(G)** Iso3 (Complex), and **(H)** Iso4 (Complex).

We also generated the porcupine plot corresponding to PC1 displaying the directions of movements (see Figure 7). In Figure 7, the arrow corresponds to the direction of the movement, while the length of the arrow represents the movement’s strength. Except for the terminal region, most regions showed stability or minor movements. However, the crown region or the carbohydrate-binding region displayed the least movement for apo and complexes except for Iso1. In the case of apo Iso1, DL2 showed a movement toward the binding region. However, in the case of the bound state, the direction of the movement was completely reversed. A similar trend was observed for apo Iso3. Apart from the DL2 loop, some parts of the CL3 loop showed upward movements or toward the crown region. However, this cysteine–cysteine loop region is far from the binding region. So, the flexible movement of this region may not affect the glycan binding.

**FIGURE 8:**
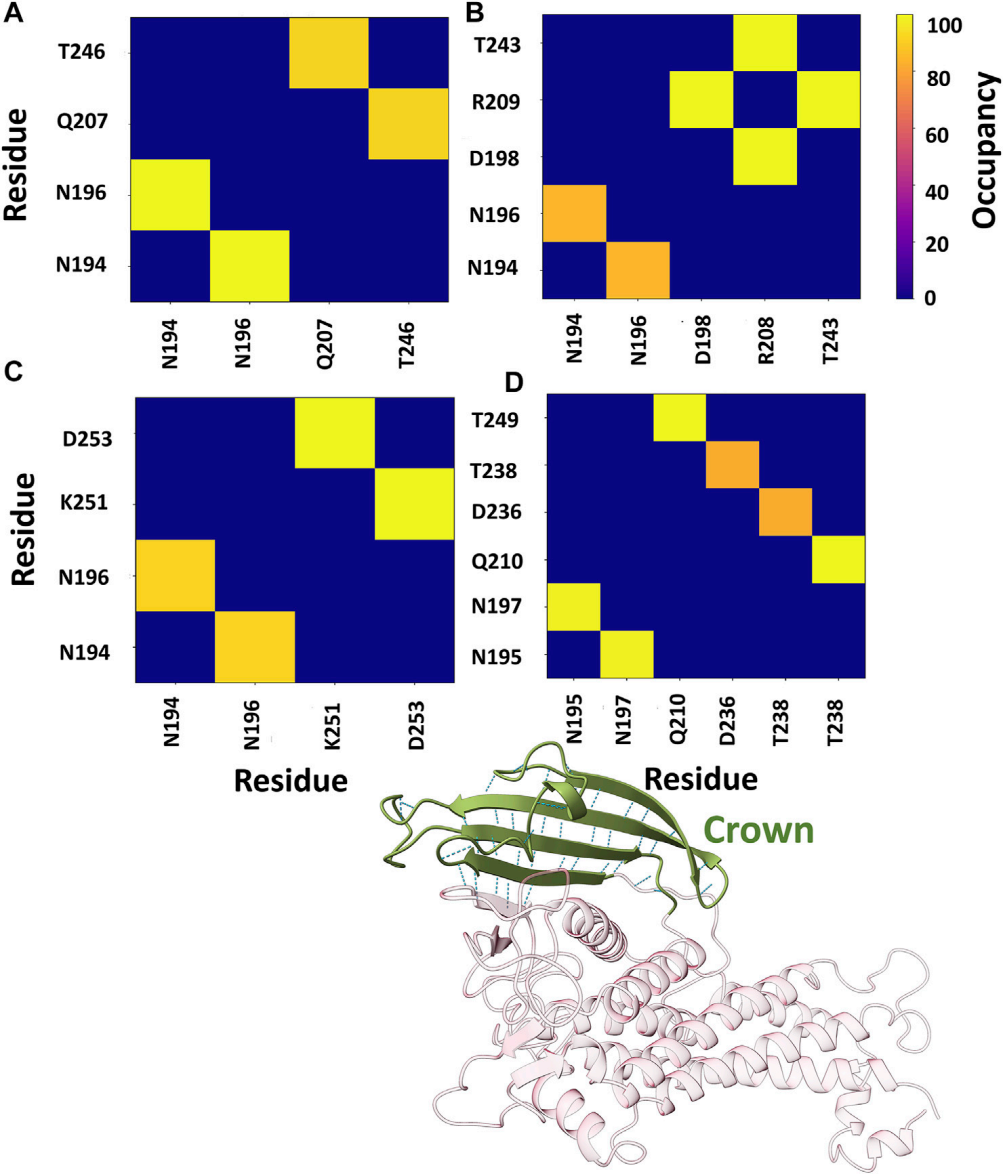
Heatmap representation of the possible H-bond paths of the crown region of BabA using Bridge2 (Siemers and Bondar, 2021). Here, the residue pairs with more than 70% occupancy were listed. **(A)** Iso1 (Complex), **(B)** Iso2 (Complex), **(C)** Iso3 (Complex), and **(D)** Iso4 (Complex).

Further, the structural stability of the crown region was investigated by estimating the number of intra-residue hydrogen bonds (H-bonds). We evaluated the occupancy of H-bonds of the crown region using the Bridge2 software (Siemers and Bondar, 2021). The H-bonds heatmap corresponding to the crown region is shown in Figure 8, where only H-bonds with more than 70% occupancy are listed. For all cases, two to three stable H-bonds were observed, which may determine the structural integrity of the region. The key residues obtained from this analysis are primarily located in the DL2 loop and its surrounding region.

Furthermore, we also estimated the joint occupancy of H-bonds, which reports true when all the estimated H-bonds are present and false otherwise (Siemers and Bondar, 2021). The joint occupancy for Iso2 was 83.9%, the highest among all isoforms (Iso1 ∼61.5%, Iso3 ∼56.3%, and Iso4 ∼57.3%). Despite the high occupancy of different hydrogen bond pairs, the DL2 region was more flexible across several isoforms of BabA.

Furthermore, to provide a deeper understanding of the dynamicity of significant loop regions, an inspection of loop regions was conducted by estimating the RMSD. The time evolution of all three cysteine–cysteine loops (CL1, CL2, and CL3) RMSD was estimated over all three MD runs and shown in Supplementary Figures S9–S11. In all three replicas, no major conformational changes were observed. This behavior was also true for the diversity loop 1 (DL1), as shown in Supplementary Figure S12. We performed the backbone dihedral angles principal component analysis (dPCA) to extract more information about the conformational changes. CL1 and CL3 loops, away from the binding region, showed a moderate conformational basin across all four isoforms (see Supplementary Figures S13 and 14). For apo structures, the principal minima of CL1 were separated from the rest of the conformational spaces by a high energy barrier. However, after the complex formation, these regions were connected by the shallow free energy barrier. On the other hand, for CL3, the conformation variability remains unchanged before and after the complex formation. As these regions are far apart from the carbohydrate-binding site, the dynamics of these regions may not interfere with Le^b^ binding.

**FIGURE 9:**
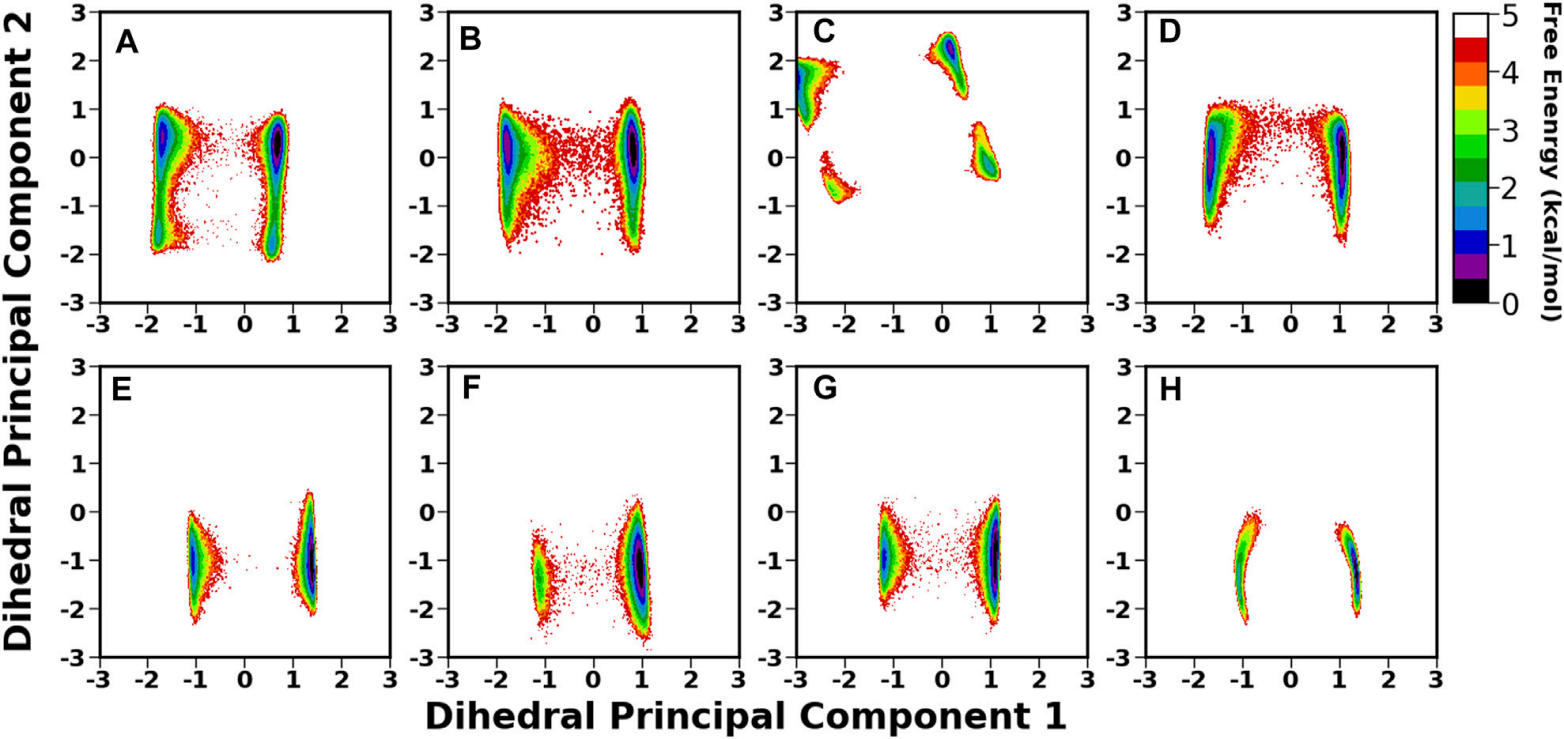
2D free energy surface (FES) generated using the first two dihedral PCA (dPCA) components of the CL2 region. **(A)** Iso1 (Apo), **(B)** Iso2 (Apo), **(C)** Iso3 (Apo), **(D)** Iso4 (Apo), **(E)** Iso1 (Complex), **(F)** Iso2 (Complex), **(G)** Iso3 (Complex), and **(H)** Iso4 (Complex).

Among all the cysteine-cysteine and diversity loops, the major loops are CL2, DL1, and DL2, which play a crucial role in ligand binding (Moonens et al., 2016). For CL2, the free energy surface was constructed using the first two dPCs, as shown in Figure 9. For all the cases, the free energy surface was characterized by two equiprobable minima, separated by a high energy barrier (Figure 9). However, the minima regions shrunk in the bound state compared to the apo form. The key change was observed in Iso3, where the apo BabA was sampled over four different areas separated by a high energy barrier. However, this intrinsic flexibility diminished after binding with Le^b^, indicating a better ligand binding phenomenon. Furthermore, to investigate the conformational dynamics of DL1 and DL2 in detail, dPCA was conducted, and the resultant FES is shown in Supplementary Figures S15 and S26. The DL1 loop showed a very stable profile throughout the replica runs, as is evident from the time series of RMSD (Supplementary Figure S12). However, flexibility was observed for all apo and complexes. Among all isoforms, Iso3 showed a more stable conformational space, and the corresponding free energy surface was characterized by two principal minima in the apo and complex forms. It should be noted here that the location of the minima region shifted after the complexation.

**FIGURE 10:**
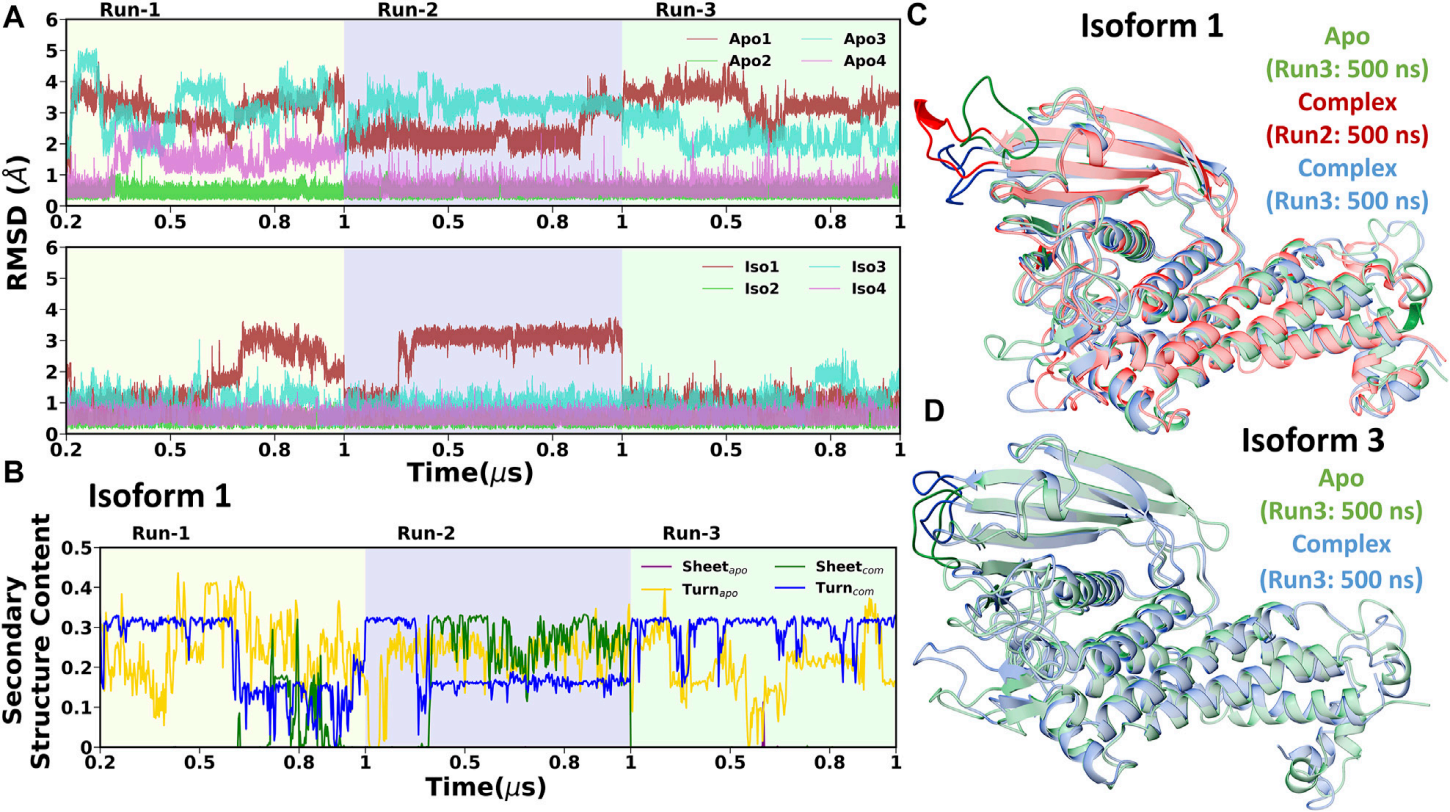
**(A)** Time evolution of root mean square deviation (RMSD) of DL2 loop over three replicas run. Apo and complex cases are shown in the first and second column, respectively. **(B)** Time evolution of secondary structure content (Beta sheet and turn) of DL2 segment in Iso1 (Apo and complex). Structural superimposition of BabA from the mentioned regions for **(C)** Iso1 and **(D)** Iso3.

In DL2, the flexibility was observed only in cases of apo forms of Iso1 and Iso3, which decreased after the binding of Le^b^, as evident from Supplementary Figure S16A and C (for apo cases) and Supplementary Figure S16E and G (for complexes). Similar behavior was observed from the RMSD plot of the DL2 loop in apo and complex forms (see Figure 10A). In apo cases, all three replica runs showed flexibility over the entire 1.0 *µs* for Iso1 and Iso3. The RMSF analysis (Figure 3) also supports the same observation.

To investigate the reason behind the shift in RMSD, we conducted the DSSP analysis using the *cpptraj* module of AmberTools19 (Roe and Cheatham, 2013), where specifically a change in *β*-sheet and turn was observed. The time evolution of the secondary structure in Iso1 for three replica runs is shown in Figure 10B. The flexible behavior of the DL2 loop in apo cases resonates with the percentage change in the turn of this segment. However, DL2 did not form any *β*-sheet in the apo simulation of BabA isoforms. In contrast, after the complexation with Le^b^, a change in coordination of sheet and turn was observed, agreeing with the RMSD profile. Specifically, when the RMSD was stable, a steady percentage of turn was observed, which diminished with the fluctuation. Contrarily, we observed the appearance of *β*-sheet with the increase in RMSD. Therefore the occurrence of *β*-sheet was inversely proportional to the decrease in the percentage of turn in the loop structure. The location of the *β*-sheet could be seen in the representative structure from the respective segment of RMSD (Figure 10C, Iso1). Similarly, the 3D conformation was also shown for Iso3 to indicate the change in the structure of the DL2 loop region (Figure 10D).

Overall, our study revealed that the intrinsic flexible nature of Iso1 persists after the glycan binding. In the case of apo Iso3, the flexibility of the crown region was relatively higher than the other three isoforms, which got drastically reduced after the complex formation. It indicates a stable Le^b^ binding for Iso3 compared to the other isoforms.

### The Binding Energetics Behind the Molecular Recognition of Le^b^ Toward Variants of BabA

**FIGURE 11:**
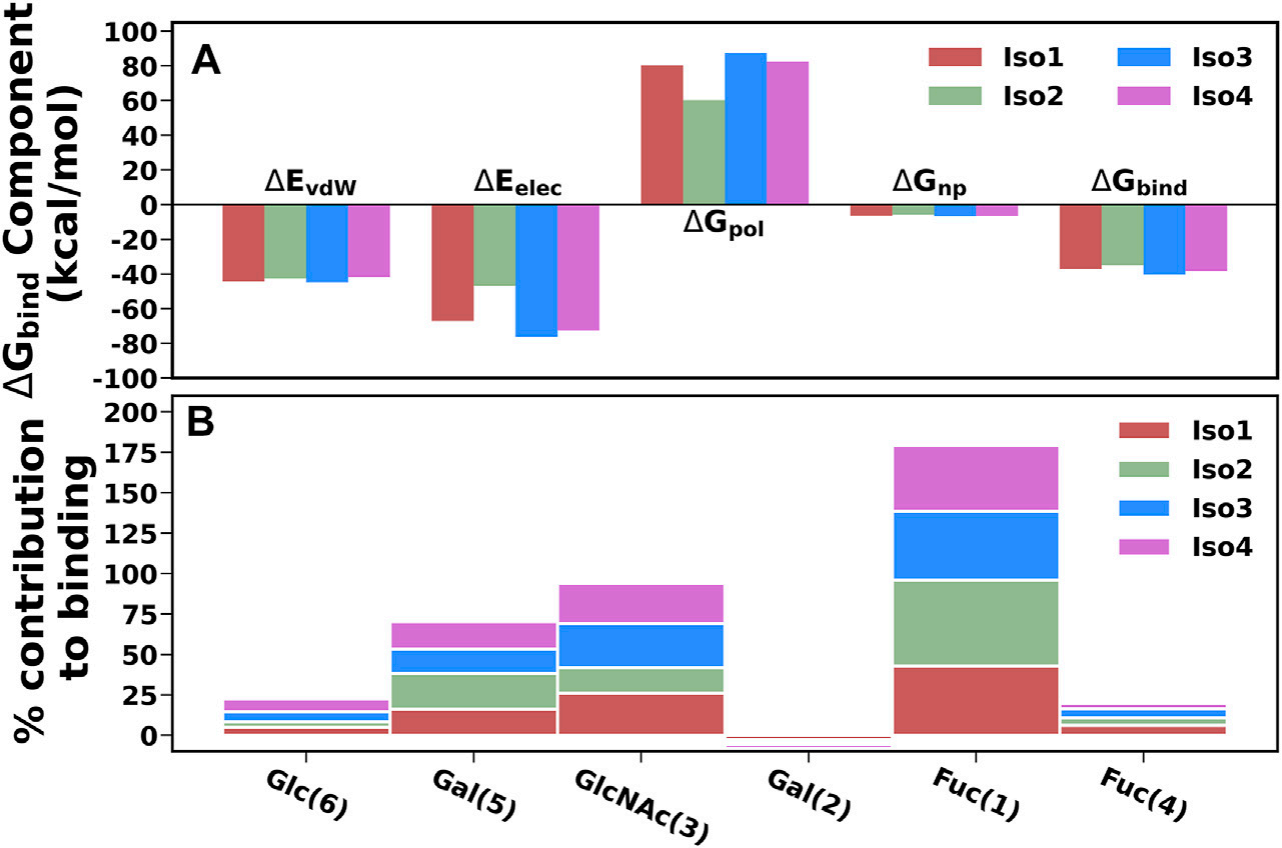
**(A)** Energetic components of binding free energy for all four complexes using MM/GBSA scheme. **(B)** Percentage contribution of each monosaccharide for all four isoform complexes.

To elucidate the binding mechanism of Le^b^ against variants of BabA, we calculated the binding free energy for each complex and its different components (see Table 2). We calculated the average binding free energy over the three replica runs and found −37.70, −34.96, −40.26, and −38.28 kcal/mol for Iso1, Iso2, Iso3, and Iso4, respectively. Overall, it suggests that Le^b^ has a stronger affinity toward Iso3 of BabA than the other three isoforms. In contrast, Iso2 showed the lowest affinity for Le^b^ (see Figure 11A). It is to be noted that the electrostatic contribution (ΔE_elec_) was more favorable than the van der Waals (ΔE_vdW_) interactions for all complexes. ΔE_elec_ varied between −46.72 kcal/mol and −76.25 kcal/mol, while ΔE_vdW_ varied between −41.77 kcal/mol and −44.71 kcal/mol. The total favorable interaction energy (ΔE_MM_), which comprises ΔE_vdW_ and ΔE_elec,_ was found to be more favorable for Iso3 (ΔE_MM_ = −120.96 kcal/mol) than other complexes. The lowest value was obtained for Iso2 (ΔE_MM_ = -89.65 kcal/mol). In the case of Iso3, both ΔE_vdW_ (−44.71 kcal/mol) and ΔE_elec_ (−76.25 kcal/mol) were more favorable than other complexes. Although ΔE_elec_ was more favorable to the complexation compared to ΔE_vdW,_ the net electrostatic component, i.e., the sum of intermolecular electrostatic and polar solvation free energy (ΔE_elec_+ ΔG_pol_), was unfavorable to Le^b^ binding for all complexes. It suggests that the intermolecular van der Waals interactions mainly governed the binding of Le^b^. Overall, for Iso3, both components (ΔE_elec_ and ΔE_vdW_) favor the BabA–Le^b^ complexation the most compared to other isoforms, resulting in a better affinity for Le^b^.

**TABLE 2:** The binding free energies of all four complexes along with their different energy components from the MM/GBSA scheme in kcal/mol. Standard deviation is given in parentheses.

System	—	ΔE_vdW_	ΔE_elec_	ΔG_pol_	ΔG_np_	ΔE_MM_ ^a^	ΔG_solv_ ^b^	ΔG_Total_ ^c^
**Iso1**	**Run1**	−43.96	−70.22	82.48	−6.52	−114.18	75.96	−38.22
	**Run2**	−46.35	−55.38	71.97	−6.39	−101.73	65.16	−36.16
	**Run3**	−42.85	−75.59	86.22	−6.50	−118.44	79.71	−38.73
	**Average**	−44.39 (1.79)	−67.06 (10.49)	80.22 (7.39)	−6.47 (0.07)	−111.45 (8.68)	73.61 (7.55)	**−37.70 (1.36)**
**Iso2**	**Run1**	−42.74	−46.10	60.25	−5.62	−88.84	54.62	−34.22
	**Run2**	−43.50	−46.70	60.50	−5.80	−90.19	54.70	−35.49
	**Run3**	−41.73	−47.35	59.83	−5.91	−89.91	53.91	−35.16
	**Average**	−42.66 (0.89)	−46.72 (0.63)	60.19 (0.34)	−5.78 (0.15)	−89.65 (0.71)	54.41 (0.43)	**−34.96 (0.66)**
**Iso3**	**Run1**	−45.79	−80.03	91.56	−6.95	−125.82	84.61	−41.21
	**Run2**	−42.88	−68.63	80.19	−6.43	−111.50	73.75	−37.75
	**Run3**	−45.47	−80.09	90.59	−6.85	−125.56	83.74	−41.82
	**Average**	−44.71 (1.59)	−76.25 (6.59)	87.45 (6.30)	−6.74 (0.28)	−120.96 (8.19)	80.70 (6.03)	**−40.26 (2.19)**
**Iso4**	**Run1**	−42.27	−68.90	81.08	−6.48	−111.16	74.61	−36.56
	**Run2**	−40.95	−78.03	84.95	−6.66	−118.98	78.29	−40.68
	**Run3**	−42.08	−70.40	81.48	−6.60	−112.48	74.89	−37.60
	**Average**	−41.77 (0.71)	−72.44 (4.89)	82.50 (2.12)	−6.58 (0.09)	−114.21 (4.19)	75.93 (2.05)	**−38.28 (2.14)**

a ΔE_vdW +_ ΔE_elec_.

b ΔG_pol +_ ΔG_np_.

c ΔE_vdW +_ ΔE_elec +_ ΔG_pol +_ ΔG_np_.

ΔE_vdW_, van der Waals Contribution; ΔE_elec_, electrostatics contribution; ΔG_pol_, polar contribution; ΔG_np_, non-polar contribution; ΔE_MM_, Molecular mechanics; ΔG_solv_, Total solvation, ΔG_Total_, Total binding free energy.

It is worth noting here that the binding free energy across the triplicate runs varied to a certain extent for all except Iso2. To investigate the origin of variation, we calculated the RMSF of the bound Le^b^ (Supplementary Figure S17). It is evident from Supplementary Figure S17 that in the case of Iso2, Le^b^ showed the least flexibility compared to the other three isoforms. Consequently, Iso2 showed almost similar binding free energy in all three replicas. However, Iso2 showed the lowest binding free energy among all four cases. It may indicate the fluctuation of a few monosaccharides can influence the protein-glycan binding free energy. However, the key residue, such as fucose, displayed the least flexibility across all four isoforms that govern the protein-glycan recognition.

The percentage contribution of each monosaccharide to the total binding was plotted and shown in Figure 11B. Fuc (1) and GlcNAc(3) contributed more favorably to binding than other monosaccharides for all complexes. Furthermore, our study revealed that Fuc (1) contributed ∼40–53% to the total binding free energy among monosaccharides in each isoform, and GlcNAc(3) contributed ∼15–27%. In Iso2, Fuc (1) contributed the highest among other isoforms, accounting for 53%, while the contribution of GlcNAc(3) was the lowest (∼15%) compared to other isoforms. It agrees with a previous study where it was reported that Fuc and GlcNAc contributed more toward the total binding free energy that favors the cholera toxin (CT)/LewisY complexation (Roy et al., 2020a).

### Per-Residue Decomposition of ΔG_bind_

**FIGURE 12:**
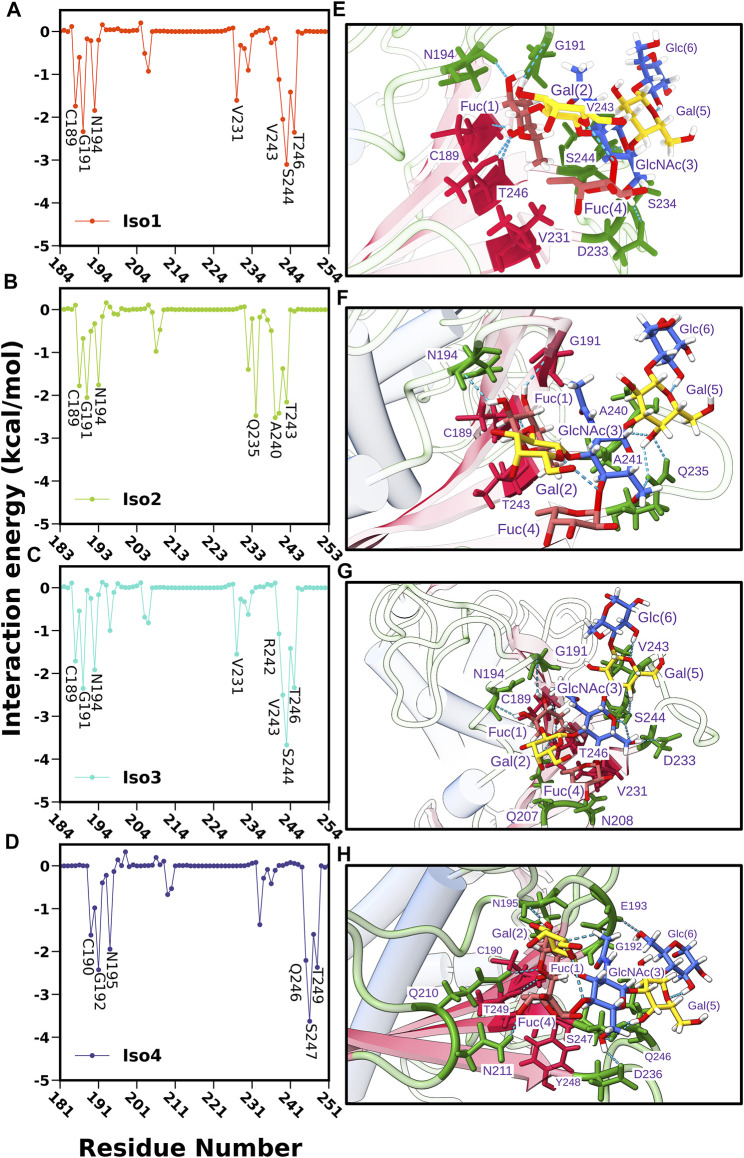
Per residue-wise contribution to the binding free energy of BabA and Le^b^ complexes: **(A)** Iso1, **(B)** Iso2, **(C)** Iso3, and **(D)** Iso4. Interaction profile of glycan and protein in the binding site location where glycans and proteins are shown in ball and stick model and ribbon representation, respectively, **(E)** Iso1, **(F)** Iso2, **(G)** Iso3, and **(H)** Iso4.

Next, we computed the contribution of each amino acid of BabA to the total binding free energy to find out the hotspot residues. All amino acids contributing more than −1.5 kcal/mol of energy are listed in Supplementary Table S1 and graphically shown in Figure 12. We noted that the highest contributing residues toward Le^b^ binding (>−3.5 kcal/mol) come from Iso3 and Iso4 in agreement with the binding energetics given previously, especially the residue S440 from Iso3 and S247 from Iso4. Similarly, S244 of Iso1 contributed high in the binding with a -3.10 kcal/mol value. In contrast, in the case of Iso2, the highest contributing residue, A240, showed a contribution of -2.52 kcal/mol, which also agrees with the lesser binding affinity than others toward Le^b^. The other contributing residues involved in the binding included T246, G191, V243, N194 (Iso1), A240, Q235, A241, T243, G191 (Iso2), V243, G191, T246, N194 (Iso3), and G192, T249, Q246, N195 (Iso4). Overall, these results suggest that primary interacting residues for recognizing Le^b^ come from CL2 (G191 and N194) and DL2 (V243, Q235, and S244) loops, which agrees with the above mentioned RMSF and free energy surface analysis. Moreover, these results are consistent with the experimental findings in explaining the significant role of CL2 and DL2 loops in recognizing glycans for the attachment of BabA of *H. pylori* (Moonens et al., 2016).

In addition, we calculated the contributions of each monosaccharide to ΔG_bind_ for all complexes and listed them in Supplementary Table S2. Fuc (1) contributed more favorably to the protein-glycan binding than other monosaccharides for all complexes. The contribution of Fuc (1) ranged from -7.65 to -8.33 kcal/mol and was more favorable for Iso3 and Iso4 than Iso1 and Iso2. In addition, GlcNAc also contributed more favorably to the binding. The contribution of GlcNAc was found to be the highest for Iso3 (-5.33 kcal/mol), followed by Iso4 (-5.11 kcal/mol), Iso1 (−4.77 kcal/mol), and Iso2 (−2.25 kcal/mol). However, in Iso2, Gal (1) contributed more significantly (−3.19 kcal/mol) than other monosaccharides. Therefore, it suggests that GlcNAc is the next significant monosaccharide favoring the association between Le^b^ and BabA. Furthermore, it is revealed from Supplementary Table S2 that the electrostatic contribution (*T*_ele_) of Fuc (1) to the total binding was more favorable than *T*_vdW_ for each complex ranging from -12.04 kcal/mol to -19.83 kcal/mol. However, *T*_ele_ was overcompensated by the disfavourable polar solvation of energy (*T*_GB_) in each complex. Therefore, the key contributing component of Fuc (1) was vdW (*T*_vdW_) which favored the binding.

Subsequently, we computed the H-bond occupancy formed between Le^b^ and BabA for all isoforms, as listed in Supplementary Table S3. Supplementary Figure S18 shows that isoforms 1, 3, and 4 showed high H-bond frequency compared to Iso2. The critical BabA–Le^b^ H-bond interactions are noted in Supplementary Table S3. It suggests that Fuc (1) formed multiple H-bonds with BabA residues (G191, T246, N194, and C189), with the occupancy varying between ∼67 and ∼90% for Iso1, while it ranged between ∼62 and ∼88% for Iso3. Similarly, T249, C190, G192, and N195 from Iso4 interacted with Fuc (1) *via* H-bond, with the occupancy varying between ∼56 and ∼86%. In the case of Iso2, Fuc (1) formed H-bond with C156, N161, G158, and T210, with an occupancy ranging from ∼40 to ∼87%. Compared to other isoforms, the number of residues forming H-bonds with Le^b^ was less in Iso2. It is evident from Supplementary Figure S18A that the average number of H-bonds in isoforms 1, 2, and 3 was estimated as 8, while on average, 6 H-bonds were observed for Iso2.

Further, we computed the hydrophobic contacts between BabA–Le^b^ as shown in Supplementary Figure S18B. The average value of the hydrophobic contact was the highest for Iso2 (∼12), while for other isoforms, on average, five to six contacts were formed. Altogether, it suggests that the electrostatic interactions favored the association between Le^b^ and BabA for isoforms 1, 3, and 4, while the hydrophobic interaction favors the complexation in Iso2.

The abovementioned analysis was complemented by plotting the interaction diagram of BabA/Le^b^ for all four complexes (see Supplementary Figure S19). As seen from Supplementary Figure S19C, in Iso3, Fuc (1) formed H-bond interactions with G191, C189, T246, and N194. Similarly, GluNAc(3) formed H-bonds with D233, Gal (5) with S244, and Fuc (4) with N208. The spike representation was used to depict the hydrophobic interactions. Overall, Supplementary Figure S19A–D indicate fewer interactions in Iso2 than other isoforms, which agrees with the aforementioned binding affinity. Overall, the interaction profile suggests a strong hydrogen bonding and hydrophobic interactions between BabA and Le^b^ for Iso3 compared to other isoforms of the BabA family, which could unravel the molecular specificity of hexasaccharide Le^b^ against isoforms of *H. pylori* adhesin BabA.

### Influence of Acidic Environment in the Binding Mechanism

Finally, we estimated the binding affinity of Iso1 (17875 strain) at acidic pH (4.5) to elucidate the effect of pH on glycan binding. The RMSD of BabA for both runs was estimated and shown in Supplementary Figure S20. Both runs attained equilibrium after 100 ns, and the last 150 ns were used to calculate the binding free energy using the MM/GBSA protocol. The binding affinity of Iso1 against Le^b^ in the acidic condition was estimated as −34.20 kcal/mol, which is ∼4 kcal/mol lower than what was evaluated in neutral solution (−37.70 kcal/mol). This finding was also supported by the recent experimental results where BabA senses the change in pH in the environment (Bugaytsova et al., 2017). In acidic conditions, the binding affinity of BabA against Le^b^ was dramatically reduced for the strain 17875, agreeing with our MM/GBSA calculation.

Details of the binding free energy and its components are listed in Supplementary Table S4. An increase in the polar solvation energy and decrease in the van der Waals component mainly lowered the binding free energy. However, Iso1 lacks the key salt bridge interaction between D198 and R207, which plays a significant role in acid sensitivity; still, it senses the change in environment as expected by the experimental findings (Hatakeyama, 2017). The short α helix, or the key-coil (residue 199-202) region located in the DL1 loop, is present in Iso1. However, the central residue in the key-coil region for acid sensitivity and binding affinity, i.e., P199, was not present in the 17875 strain, which may reduce the sensitivity against the pH change (Bugaytsova et al., 2017). The critical residues involved in the binding are shown in Supplementary Figure S21, indicating a similar pattern relative to the neutral condition. Irrespective of pH, the key residues are the same, with a minor variation determining the strength of the binding. In acidic conditions, direct contributions from the amino acids of the key-coil region were not observed.

## Conclusion

Herein, we performed all-atom multiple replicas molecular dynamics simulations to elucidate the recognition mechanism of the O blood group antigen (Le^b^) by BabA of *H. pylori*. This study involved the O specialist (Iso3) and ABO generalist strains of *H. pylori*. We investigated the conformational preferences of key amino acids present in the crown region of BabA upon Le^b^ binding. Our study revealed no significant change in the flexibility of Le^b^ in the free and BabA-bound states, which was evident from the ring puckering profile. In addition, we showed that the difference in the recognition process between Le^b^ and BabA was due to the distinct movement of loops (CL2, DL1, and DL2) near the glycan-binding sites. Among all isoforms, Iso1 (strain 17875) showed the maximum motions near the DL2 loop, which persisted after the glycan binding. Interestingly, despite the flexibility of the DL2 loop, glycan stays close to the binding pocket due to the changes in secondary conformations. For Iso3 (strain S831), the outward movement of the DL2 loop vanished upon ligand binding, stipulating a better binding partner. Furthermore, structural changes in CL2 were observed for the S831 strain (Iso3) upon glycan binding. This implies that the recognition process must rely on the receptor’s conformational dynamics, which were investigated by characterizing several key loops, such as CL2, DL1, and DL2. Estimating the conformational flexibility of the crown region indicates the stable 2–3 H-bonds located mainly in the DL2 region, which determines the binding region’s structural integrity. In our study, the flexibility of the CL2 loop was drastically reduced for Iso3, which indicates an increased binding affinity. The higher flexibility of the DL2 loop in the bound state for Iso1 results from the interplay between beta-sheet and turn conformations.

To quantify the conformational changes with the binding affinity of Le^b^, we estimated the binding free energy using the MM/GBSA method. It ranks Iso3 as a strong binding partner, whereas Iso2 shows the least binding affinity among all four isoforms. In the case of Iso3, the stabilization of the critical loops helps achieve a better binding affinity than other isoforms. As this strain is O specialist in nature, better binding supports the experimental characteristics of this strain. The Fuc (1) residue, linked by the *α*1-2 linkage, provides a significant contribution to the binding. The role of this specific fucose was also blended with the crystallographic information of the BabA–Le^b^ interaction (Moonens et al., 2016). In addition, critical residues involved in the binding, i.e., G191, N194, V243, Q235, and S244, mainly lie in the CL2 and DL2 loop regions implying the importance of these regions in the recognition process. Finally, we investigated the effect of pH on the BabA–Le^b^ association by performing constant pH MD simulations, which revealed a reduction in the binding free energy in an acidic environment. Overall, our study provides a detailed understanding of the interaction of O blood group Lewis hexasaccharide across different isoforms of BabA.

## Data Availability

The raw data supporting the conclusions of this article will be made available by the authors, without undue reservation.
