# Supplementary material for: Comparative Structural Dynamics of Isoforms of Helicobacter pylori Adhesin BabA Bound to Lewis b Hexasaccharide via Multiple Replica Molecular Dynamics Simulations
Source: Front Mol Biosci. 2022 May 2;9:852895. doi: 10.3389/fmolb.2022.852895 (PMC9108286; doi:10.3389/fmolb.2022.852895)
Supplement: Supplementary file 1 [file DataSheet1.docx]

**Comparative structural dynamics of isoforms of Helicobacter pylori adhesin BabA bound to Lewis b hexasaccharide via multiple replica molecular dynamics simulations**

Rajarshi Roy**^ǂ^**, Nisha Amarnath Jonniya**^ǂ^**, Md Fulbabu Sk, Parimal Kar^*^

Department of Biosciences and Biomedical Engineering, Indian Institute of Technology Indore,

Khandwa Road, Madhya Pradesh 453552, India

^ǂ^Authors contributed equally

^*^Corresponding Author: Parimal Kar | Email: [parimal@itii.ac.in](mailto:parimal@itii.ac.in)

**Table S1:** The per residual decomposition of free energy from variants of BabA depicting the significant residues in the interaction with Le^b^.

| **Residue** | ***T_vdw_*** | ***T_ele_*** | ***T_pol_*** | ***T_np_*** | ***T_side_*** | ***T_back_*** | ***T_total_*** |
| --- | --- | --- | --- | --- | --- | --- | --- |
| **Iso1** | | | | | | | |
| **S244** | | | | | | | |
| **Run1** | -2.82 | -3.31 | 3.23 | -0.42 | -3.03 | -0.29 | -3.32 |
| **Run2** | -3.23 | -1.16 | 2.44 | -0.36 | -2.25 | -0.06 | -2.31 |
| **Run3** | -2.67 | -4.24 | 3.66 | -0.42 | -3.45 | -0.22 | -3.67 |
| **Average** | -2.91 | -2.91 | 3.11 | -0.40 | -2.91 | -0.19 | -3.10 |
| **T246** | | | | | | | |
| **Run1** | -0.81 | -3.31 | 1.89 | -0.11 | -2.17 | -0.17 | -2.34 |
| **Run2** | -0.93 | -3.45 | 2.13 | -0.12 | -2.08 | -0.30 | -2.38 |
| **Run3** | -0.81 | -3.22 | 1.79 | -0.10 | -2.21 | -0.13 | -2.34 |
| **Average** | -0.85 | -3.33 | 1.94 | -0.11 | -2.15 | -0.20 | -2.35 |
| **G191** | | | | | | | |
| **Run1** | -0.85 | -2.72 | 1.46 | -0.17 | -0.26 | -2.01 | -2.28 |
| **Run2** | -0.54 | -3.34 | 1.61 | -0.13 | -0.22 | -2.19 | -2.41 |
| **Run3** | -0.96 | -2.76 | 1.57 | -0.18 | -0.28 | -2.04 | -2.32 |
| **Average** | -0.78 | -2.94 | 1.55 | -0.16 | -0.25 | -2.08 | -2.33 |
| **V243** | | | | | | | |
| **Run1** | -2.41 | -1.01 | 1.22 | -0.28 | -1.92 | -0.57 | -2.48 |
| **Run2** | -1.47 | -0.47 | 0.96 | -0.18 | -1.15 | -0.01 | -1.17 |
| **Run3** | -2.42 | -0.99 | 1.21 | -0.30 | -1.97 | -0.53 | -2.50 |
| **Average** | -2.10 | -0.82 | 1.13 | -0.25 | -1.68 | -0.37 | -2.05 |
| **N194** | | | | | | | |
| **Run1** | -0.85 | -3.06 | 2.12 | -0.17 | -0.56 | -1.41 | -1.96 |
| **Run2** | -0.89 | -2.46 | 1.91 | -0.16 | -0.54 | -1.05 | -1.59 |
| **Run3** | -0.75 | -3.21 | 2.15 | -0.16 | -0.51 | -1.47 | -1.98 |
| **Average** | -0.83 | -2.91 | 2.06 | -0.16 | -0.54 | -1.31 | -1.84 |
| **C189** | | | | | | | |
| **Run1** | -0.53 | -1.30 | 0.16 | -0.03 | -0.28 | -1.42 | -1.69 |
| **Run2** | -0.67 | -1.46 | 0.49 | -0.04 | -0.38 | -1.30 | -1.68 |
| **Run3** | -0.49 | -1.41 | 0.08 | -0.03 | -0.30 | -1.56 | -1.86 |
| **Average** | -0.56 | -1.39 | 0.24 | -0.03 | -0.32 | -1.43 | -1.74 |
| **V231** | | | | | | | |
| **Run1** | -1.59 | -0.49 | 0.68 | -0.22 | -1.71 | 0.09 | -1.62 |
| **Run2** | -1.71 | -0.34 | 0.54 | -0.18 | -1.82 | 0.14 | -1.68 |
| **Run3** | -1.50 | -0.55 | 0.74 | -0.21 | -1.61 | 0.09 | -1.52 |
| **Average** | -1.60 | -0.46 | 0.65 | -0.20 | -1.71 | 0.11 | -1.61 |
| **Iso2** | | | | | | | |
| **A240** | | | | | | | |
| **Run1** | -2.37 | -1.01 | 1.18 | -0.34 | -1.62 | -0.92 | -2.55 |
| **Run2** | -2.38 | -1.05 | 1.19 | -0.34 | -1.63 | -0.95 | -2.57 |
| **Run3** | -2.29 | -0.99 | 1.17 | -0.34 | -1.58 | -0.86 | -2.44 |
| **Average** | -2.35 | -1.02 | 1.18 | -0.34 | -1.61 | -0.91 | -2.52 |
| **Q235** | | | | | | | |
| **Run1** | -1.07 | -6.49 | 5.36 | -0.35 | -2.63 | 0.09 | -2.55 |
| **Run2** | -1.09 | -6.60 | 5.43 | -0.35 | -2.70 | 0.09 | -2.61 |
| **Run3** | -1.12 | -5.89 | 5.08 | -0.34 | -2.34 | 0.08 | -2.26 |
| **Average** | -1.09 | -6.33 | 5.29 | -0.35 | -2.56 | -0.09 | -2.47 |
| **A241** | | | | | | | |
| **Run1** | -2.79 | -0.13 | 0.84 | -0.34 | -1.89 | -0.53 | -2.42 |
| **Run2** | -2.78 | -0.14 | 0.81 | -0.34 | -1.89 | -0.55 | -2.44 |
| **Run3** | -2.75 | -0.16 | 0.86 | -0.34 | -1.85 | -0.53 | -2.39 |
| **Average** | -2.77 | -0.14 | 0.84 | -0.34 | -1.88 | -0.54 | -2.42 |
| **T243** | | | | | | | |
| **Run1** | -0.68 | -2.89 | 1.42 | -0.07 | -2.16 | -0.07 | -2.23 |
| **Run2** | -0.70 | -2.84 | 1.40 | -0.07 | -2.14 | -0.08 | -2.22 |
| **Run3** | -0.74 | -3.07 | 1.96 | -0.16 | -2.00 | -0.02 | -2.02 |
| **Average** | -0.71 | -2.93 | 1.59 | -0.10 | -2.10 | -0.0.6 | -2.16 |
| **G191** | | | | | | | |
| **Run1** | -0.91 | -2.34 | 1.39 | -0.17 | -0.27 | -1.76 | -2.03 |
| **Run2** | -0.89 | -2.35 | 1.37 | -0.17 | -0.26 | -1.77 | -2.04 |
| **Run3** | -0.74 | -2.62 | 1.43 | -0.17 | -0.26 | -1.83 | -2.09 |
| **Average** | -0.85 | -2.44 | 1.40 | -0.17 | -0.26 | -1.79 | -2.05 |
| **C189** | | | | | | | |
| **Run1** | -0.55 | -1.40 | 0.12 | -0.02 | -0.29 | -1.56 | -1.85 |
| **Run2** | -0.54 | -1.43 | 0.14 | -0.02 | -0.28 | -1.58 | -1.86 |
| **Run3** | -0.58 | -1.12 | 0.11 | -0.03 | -0.27 | -1.35 | -1.62 |
| **Average** | -0.56 | -1.32 | 0.12 | -0.02 | -0.28 | -1.50 | -1.78 |
| **N194** | | | | | | | |
| **Run1** | -1.12 | -2.29 | 1.90 | -0.12 | -0.63 | -1.00 | -1.62 |
| **Run2** | -1.15 | -2.26 | 1.88 | -0.12 | -0.63 | -1.01 | -1.64 |
| **Run3** | -0.98 | -2.96 | 2.11 | -0.17 | -0.62 | -1.38 | -2.00 |
| **Average** | -1.08 | -2.50 | 1.96 | -0.14 | -0.63 | -1.13 | -1.76 |
| **Iso3** | | | | | | | |
| **S244** | | | | | | | |
| **Run1** | -2.67 | -4.30 | 3.67 | -0.42 | -3.54 | -0.18 | -3.72 |
| **Run2** | -2.53 | -4.25 | 3.57 | -0.41 | -3.37 | -0.26 | -3.62 |
| **Run3** | -2.64 | -4.06 | 3.45 | -0.42 | -3.46 | -0.21 | -3.66 |
| **Average** | -2.61 | -4.20 | 3.56 | -0.42 | -3.46 | -0.22 | -3.67 |
| **V243** | | | | | | | |
| **Run1** | -2.51 | -0.97 | 1.22 | -0.31 | -2.03 | -0.53 | -2.56 |
| **Run2** | -2.21 | -0.93 | 1.13 | -0.34 | -2.00 | -0.34 | -2.35 |
| **Run3** | -2.51 | -0.98 | 1.21 | -0.32 | -2.07 | -0.54 | -2.61 |
| **Average** | -2.41 | -0.96 | 1.19 | -0.32 | -2.03 | -0.47 | -2.50 |
| **G191** | | | | | | | |
| **Run1** | -0.87 | -2.91 | 1.58 | -0.16 | -0.26 | -2.10 | -2.36 |
| **Run2** | -0.98 | -3.01 | 1.82 | -0.18 | -0.30 | -2.05 | -2.35 |
| **Run3** | -0.93 | -2.64 | 1.41 | -0.17 | -0.27 | -2.06 | -2.33 |
| **Average** | -0.93 | -2.85 | 1.60 | -0.17 | -0.28 | -2.07 | -2.35 |
| **T246** | | | | | | | |
| **Run1** | -0.83 | -3.03 | 1.77 | -0.12 | -2.06 | -0.14 | -2.20 |
| **Run2** | -0.80 | -3.24 | 1.67 | -0.09 | -2.25 | -0.22 | -2.47 |
| **Run3** | -0.81 | -3.07 | 1.65 | -0.09 | -2.19 | -0.14 | -2.32 |
| **Average** | -0.81 | -3.11 | 1.70 | -0.10 | -2.17 | -0.17 | -2.33 |
| **N194** | | | | | | | |
| **Run1** | -0.85 | -2.74 | 1.92 | -0.12 | -0.49 | -1.30 | -1.79 |
| **Run2** | -0.82 | -2.69 | 1.71 | -0.10 | -0.44 | -1.46 | -1.90 |
| **Run3** | -0.84 | -3.07 | 1.96 | -0.11 | -0.52 | -1.54 | -2.06 |
| **Average** | -0.84 | -2.83 | 1.86 | -0.11 | -0.48 | -1.43 | -1.92 |
| **C189** | | | | | | | |
| **Run1** | -0.59 | -1.26 | 0.12 | -0.03 | -0.30 | -1.46 | -1.76 |
| **Run2** | -0.58 | -0.99 | 0.04 | -0.04 | -0.23 | -1.34 | -1.57 |
| **Run3** | -0.60 | -1.34 | 0.17 | -0.03 | -0.31 | -1.49 | -1.80 |
| **Average** | -0.59 | -1.20 | 0.11 | -0.03 | -0.28 | -1.43 | -1.71 |
| **V231** | | | | | | | |
| **Run1** | -1.52 | -0.57 | 0.76 | -0.20 | -1.63 | 0.09 | -1.54 |
| **Run2** | -1.59 | -0.55 | 0.74 | -0.20 | -1.69 | 0.09 | -1.60 |
| **Run3** | -1.50 | -0.53 | 0.72 | -0.19 | -1.60 | 0.10 | -1.51 |
| **Average** | -1.54 | -0.55 | 0.74 | -0.20 | -1.64 | 0.09 | -1.55 |
| **Iso4** | | | | | | | |
| **S247** | | | | | | | |
| **Run1** | -2.60 | -4.28 | 3.64 | -0.43 | -3.32 | -0.35 | -3.67 |
| **Run2** | -2.65 | -4.15 | 3.68 | -0.43 | -3.20 | -0.34 | -3.54 |
| **Run3** | -2.57 | -4.31 | 3.65 | -0.43 | -3.37 | -0.30 | -3.66 |
| **Average** | -2.61 | -4.25 | 3.66 | -0.43 | -3.30 | -0.33 | -3.62 |
| **G192** | | | | | | | |
| **Run1** | -0.99 | -2.69 | 1.47 | -0.16 | -0.29 | -2.07 | -2.36 |
| **Run2** | -1.08 | -2.96 | 1.65 | -0.14 | -0.32 | -2.21 | -2.53 |
| **Run3** | -0.99 | -2.86 | 1.61 | -0.15 | -0.30 | -2.09 | -2.39 |
| **Average** | -1.02 | -2.84 | 1.58 | -0.15 | -0.30 | -2.12 | -2.42 |
| **T249** | | | | | | | |
| **Run1** | -0.79 | -3.01 | 1.55 | -0.10 | -2.26 | -0.09 | -2.35 |
| **Run2** | -0.81 | -2.99 | 1.51 | -0.10 | -2.30 | -0.09 | -2.39 |
| **Run3** | -0.83 | -2.99 | 1.56 | -0.11 | -2.26 | -0.10 | -2.36 |
| **Average** | -0.81 | -2.99 | 1.54 | -0.10 | -2.27 | -0.09 | -2.37 |
| **Q246** | | | | | | | |
| **Run1** | -3.18 | -2.51 | 3.99 | -0.44 | -1.69 | -0.44 | -2.13 |
| **Run2** | -2.65 | -3.67 | 4.41 | -0.38 | -1.88 | -0.41 | -2.29 |
| **Run3** | -3.03 | -3.04 | 4.30 | -0.43 | -1.81 | -0.38 | -2.20 |
| **Average** | -2.95 | -3.07 | 4.23 | -0.42 | -1.79 | -0.41 | -2.21 |
| **N195** | | | | | | | |
| **Run1** | -0.75 | -3.17 | 2.14 | -0.17 | -0.47 | -1.47 | -1.94 |
| **Run2** | -0.72 | -3.25 | 2.17 | -0.16 | -0.46 | -1.50 | -1.97 |
| **Run3** | -0.74 | -3.12 | 2.10 | -0.16 | -0.47 | -1.45 | -1.92 |
| **Average** | -0.74 | -3.18 | 2.14 | -0.16 | -0.47 | -1.47 | -1.94 |
| **C190** | | | | | | | |
| **Run1** | -0.54 | -1.01 | -0.03 | -0.03 | -0.24 | -1.37 | -1.61 |
| **Run2** | -0.54 | -1.03 | -0.03 | -0.02 | -0.24 | -1.38 | -1.62 |
| **Run3** | -0.54 | -1.00 | -0.04 | -0.03 | -0.24 | -1.36 | -1.60 |
| **Average** | -0.54 | -1.01 | -0.03 | -0.03 | -0.24 | -1.37 | -1.61 |
| **Y248** | | | | | | | |
| **Run1** | -1.46 | -0.64 | 0.53 | -0.03 | -0.62 | -0.97 | -1.60 |
| **Run2** | -1.44 | -0.63 | 0.50 | -0.02 | -0.61 | -0.98 | -1.59 |
| **Run3** | -1.45 | -0.64 | 0.50 | -0.02 | -0.62 | -0.99 | -1.61 |
| **Average** | -1.45 | -0.64 | 0.51 | -0.02 | -0.62 | -0.98 | -1.60 |

**Table S2:** The per residual decomposition of free energy from the Le^b^ depicting the significant residues of glycans favouring the interaction with variants of BabA.

| **Residue** | ***T_vdw_*** | ***T_ele_*** | ***T_pol_*** | ***T_np_*** | ***T_total_*** |
| --- | --- | --- | --- | --- | --- |
| **Iso1** | | | | | |
| **Reducing End (OH)** | | | | | |
| **Run1** | -0.17 | -5.49 | 5.48 | -0.06 | -0.24 |
| **Run2** | -0.05 | -1.32 | 1.40 | -0.01 | 0.01 |
| **Run3** | -0.17 | -6.36 | 6.29 | -0.07 | -0.30 |
| **Average** | -0.13 | -4.39 | 4.39 | -0.05 | -0.18 |
| **Glc(6)** | | | | | |
| **Run1** | -2.20 | -3.46 | 4.68 | -0.50 | -1.48 |
| **Run2** | -0.92 | -0.70 | 1.82 | -0.16 | 0.04 |
| **Run3** | -2.10 | -2.98 | 4.29 | -0.47 | -1.25 |
| **Average** | -1.74 | -2.98 | 3.60 | -0.38 | -0.90 |
| **Gal(5)** | | | | | |
| **Run1** | -4.25 | -4.86 | 6.60 | -0.62 | -3.14 |
| **Run2** | -5.10 | -2.95 | 6.18 | -0.81 | -2.68 |
| **Run3** | -4.14 | -4.71 | 6.46 | -0.62 | -3.01 |
| **Average** | -4.50 | -4.17 | 6.41 | -0.68 | -2.94 |
| **GlcNAc(3)** | | | | | |
| **Run1** | -4.23 | -8.68 | 9.40 | -0.70 | -4.22 |
| **Run2** | -5.22 | -7.60 | 8.77 | -0.82 | -4.87 |
| **Run3** | -4.00 | -11.25 | 10.77 | -0.74 | -5.22 |
| **Average** | -4.48 | -9.18 | 9.65 | -0.75 | -4.77 |
| **Gal(2)** | | | | | |
| **Run1** | -1.19 | -0.26 | 1.99 | -0.07 | 0.46 |
| **Run2** | -1.25 | 0.13 | 1.76 | -0.07 | 0.57 |
| **Run3** | -1.19 | -0.31 | 2.05 | -0.07 | 0.48 |
| **Average** | -1.21 | -0.15 | 1.93 | -0.07 | 0.50 |
| **Fuc(1)** | | | | | |
| **Run1** | -7.49 | -11.58 | 12.59 | -1.35 | -7.84 |
| **Run2** | -7.61 | -12.98 | 14.28 | -1.36 | -7.67 |
| **Run3** | -7.46 | -11.56 | 12.30 | -1.35 | -8.05 |
| **Average** | -7.52 | -12.04 | 13.06 | -1.35 | -7.85 |
| **Fuc(4)** | | | | | |
| **Run1** | -2.44 | -0.77 | 2.63 | -0.49 | -1.07 |
| **Run2** | -3.03 | -2.26 | 4.49 | -0.56 | -1.36 |
| **Run3** | -2.37 | -0.63 | 2.55 | -0.48 | -0.93 |
| **Average** | -2.61 | -1.22 | 3.22 | -0.51 | -1.12 |
| **Iso2** | | | | | |
| **Reducing End (OH)** | | | | | |
| **Run1** | -0.13 | -0.75 | 0.89 | -0.02 | -0.01 |
| **Run2** | -0.14 | -0.48 | 0.61 | -0.02 | -0.02 |
| **Run3** | -0.12 | -0.34 | 0.47 | -0.02 | -0.02 |
| **Average** | -0.13 | -0.52 | 0.66 | -0.02 | -0.02 |
| **Glc(6)** | | | | | |
| **Run1** | -2.25 | -0.72 | 2.87 | -0.36 | -0.46 |
| **Run2** | -2.32 | -0.88 | 3.05 | -0.37 | -0.53 |
| **Run3** | -2.10 | -0.64 | 2.65 | -0.34 | -0.42 |
| **Average** | -2.22 | -0.75 | 2.86 | -0.36 | -0.47 |
| **Gal(5)** | | | | | |
| **Run1** | -3.51 | -5.56 | 6.32 | -0.50 | -3.25 |
| **Run2** | -3.50 | -5.80 | 6.46 | -0.49 | -3.34 |
| **Run3** | -3.43 | -4.80 | 5.75 | -0.49 | -2.97 |
| **Average** | -3.48 | -5.39 | 6.18 | -0.49 | -3.19 |
| **GlcNAc(3)** | | | | | |
| **Run1** | -3.76 | -2.02 | 4.09 | -0.56 | -2.25 |
| **Run2** | -3.83 | -1.68 | 3.79 | -0.55 | -2.27 |
| **Run3** | -3.83 | -0.20 | 2.36 | -0.55 | -2.22 |
| **Average** | -3.81 | -1.3 | 3.41 | -0.55 | -2.25 |
| **Gal(2)** | | | | | |
| **Run1** | -1.38 | -0.67 | 2.39 | -0.09 | 0.24 |
| **Run2** | -1.34 | -0.49 | 2.17 | -0.09 | 0.25 |
| **Run3** | -1.44 | -1.17 | 2.76 | -0.18 | -0.04 |
| **Average** | -1.39 | -0.78 | 2.44 | -0.12 | 0.15 |
| **Fuc(1)** | | | | | |
| **Run1** | -8.56 | -12.86 | 14.70 | -1.40 | -8.12 |
| **Run2** | -8.55 | -12.87 | 14.67 | -1.40 | -8.14 |
| **Run3** | -7.23 | -12.22 | 14.12 | -1.35 | -6.68 |
| **Average** | -8.11 | -12.65 | 14.50 | -1.38 | -7.65 |
| **Fuc(4)** | | | | | |
| **Run1** | -1.78 | -0.46 | 2.61 | -0.34 | 0.04 |
| **Run2** | -2.07 | -1.15 | 3.26 | -0.43 | -0.39 |
| **Run3** | -2.71 | -4.30 | 5.95 | -0.58 | -1.65 |
| **Average** | -2.19 | -1.97 | 3.94 | -0.45 | -0.67 |
| **Iso3** | | | | | |
| **Reducing End (OH)** | | | | | |
| **Run1** | -0.18 | -5.75 | 5.68 | -0.07 | -0.31 |
| **Run2** | -0.11 | -1.85 | 2.01 | -0.03 | 0.03 |
| **Run3** | -0.16 | -6.19 | 6.07 | -0.07 | -0.36 |
| **Average** | -0.15 | -4.60 | 4.59 | -0.06 | -0.21 |
| **Glc(6)** | | | | | |
| **Run1** | -2.26 | -4.06 | 5.11 | -0.53 | -1.74 |
| **Run2** | -1.50 | -1.91 | 3.14 | -0.32 | -0.59 |
| **Run3** | -2.12 | -3.11 | 4.34 | -0.48 | -1.38 |
| **Average** | -1.96 | -3.03 | 4.20 | -0.44 | -1.24 |
| **Gal(5)** | | | | | |
| **Run1** | -4.27 | -4.72 | 6.52 | -0.62 | -3.09 |
| **Run2** | -3.59 | -4.39 | 5.85 | -0.58 | -2.72 |
| **Run3** | -4.19 | -4.64 | 6.39 | -0.62 | -3.07 |
| **Average** | -4.02 | -4.58 | 6.25 | -0.61 | -2.96 |
| **GlcNAc(3)** | | | | | |
| **Run1** | -3.96 | -9.97 | 9.35 | -0.73 | -5.30 |
| **Run2** | -4.04 | -9.41 | 9.13 | -0.73 | -5.05 |
| **Run3** | -4.06 | -10.75 | 9.94 | -0.76 | -5.63 |
| **Average** | -4.02 | -10.04 | 9.47 | -0.74 | -5.33 |
| **Gal(2)** | | | | | |
| **Run1** | -1.56 | -0.67 | 2.73 | -0.20 | 0.30 |
| **Run2** | -1.56 | -0.35 | 2.43 | -0.15 | 0.37 |
| **Run3** | -1.52 | -0.48 | 2.63 | -0.16 | 0.46 |
| **Average** | -1.55 | -0.5 | 2.60 | -0.17 | 0.38 |
| **Fuc(1)** | | | | | |
| **Run1** | -7.93 | -12.79 | 14.25 | -1.38 | -7.85 |
| **Run2** | -8.25 | -13.90 | 14.91 | -1.40 | -8.64 |
| **Run3** | -8.22 | -12.87 | 14.01 | -1.40 | -8.47 |
| **Average** | -8.13 | -13.19 | 14.39 | -1.39 | -8.32 |
| **Fuc(4)** | | | | | |
| **Run1** | -2.74 | -2.06 | 4.13 | -0.52 | -1.19 |
| **Run2** | -2.40 | -2.50 | 4.28 | -0.46 | -1.09 |
| **Run3** | -2.46 | -1.99 | 3.92 | -0.47 | -1.00 |
| **Average** | -2.53 | -2.18 | 4.11 | -0.48 | -1.09 |
| **Iso4** | | | | | |
| **Reducing End (OH)** | | | | | |
| **Run1** | -0.20 | 4.22 | -3.90 | -0.06 | 0.05 |
| **Run2** | 0.31 | 1.46 | -3.15 | -0.14 | -1.52 |
| **Run3** | -0.05 | 3.26 | -3.72 | -0.11 | -0.63 |
| **Average** | 0.02 | 2.98 | -3.59 | -0.10 | -0.7 |
| **Glc(6)** | | | | | |
| **Run1** | -2.45 | -2.86 | 4.28 | -0.47 | -1.49 |
| **Run2** | -2.14 | -2.79 | 3.52 | -0.35 | -1.76 |
| **Run3** | -2.50 | -3.35 | 4.61 | -0.46 | -1.70 |
| **Average** | -2.36 | -3.0 | 4.14 | -0.43 | -1.65 |
| **Gal(5)** | | | | | |
| **Run1** | -3.87 | -4.88 | 6.31 | -0.57 | -3.02 |
| **Run2** | -3.90 | -7.53 | 7.83 | -0.63 | -4.23 |
| **Run3** | -3.90 | -5.33 | 6.66 | -0.59 | -3.15 |
| **Average** | -3.89 | -5.91 | 6.93 | -0.60 | -3.47 |
| **GlcNAc(3)** | | | | | |
| **Run1** | -3.62 | -2.91 | 2.08 | -0.68 | -5.12 |
| **Run2** | -3.64 | -3.47 | 2.64 | -0.68 | -5.16 |
| **Run3** | -3.54 | -2.99 | 2.13 | -0.66 | -5.06 |
| **Average** | -3.6 | -3.12 | 2.28 | -0.67 | -5.11 |
| **Gal(2)** | | | | | |
| **Run1** | -1.26 | 0.23 | 1.66 | -0.10 | 0.52 |
| **Run2** | -1.33 | 0.35 | 1.61 | -0.11 | 0.52 |
| **Run3** | -1.29 | 0.47 | 1.47 | -0.12 | 0.53 |
| **Average** | -1.29 | 0.35 | 1.58 | -0.11 | 0.52 |
| **Fuc(1)** | | | | | |
| **Run1** | -7.60 | -20.25 | 20.82 | -1.36 | -8.39 |
| **Run2** | -7.62 | -19.57 | 20.13 | -1.36 | -8.42 |
| **Run3** | -7.60 | -19.67 | 20.47 | -1.36 | -8.17 |
| **Average** | -7.61 | -19.83 | 20.47 | -1.36 | -8.33 |
| **Fuc(4)** | | | | | |
| **Run1** | -2.14 | -8.00 | 9.87 | -0.43 | -0.70 |
| **Run2** | -2.14 | -7.46 | 9.33 | -0.43 | -0.70 |
| **Run3** | -2.15 | -7.59 | 9.52 | -0.43 | -0.65 |
| **Average** | -2.14 | -7.68 | 9.57 | -0.43 | -0.68 |

**Table S3:** The hydrogen bond occupancy between BabA and Lewis sugar (Le^b^) for all four complexes calculated from the combine three replica runs. BabA residues are highlighted in bold.

| Acceptor | Donor | Distance (Å) | Angle (˚) | Occupancy (%) |
| --- | --- | --- | --- | --- |
| **Iso1** | | | | |
| **G191@O** | Fuc(1)@O3 | 2.77 | 162.96 | 89.16 |
| Fuc(1)@O4 | **T246@OG1** | 2.81 | 163.70 | 79.49 |
| **N194@O** | Fuc(1)@O2 | 2.75 | 160.50 | 74.04 |
| **C189@O** | Fuc(1)@O4 | 2.77 | 149.67 | 67.66 |
| GlcNAc(3)@O6 | **S244@OG** | 2.80 | 159.95 | 39.24 |
| **S244@OG** | Gal(5)@O4 | 2.82 | 157.13 | 37.42 |
| **D233@OD2** | GlcNAc(3)@O6 | 2.68 | 163.37 | 30.68 |
| **D233@OD1** | GlcNAc(3)@O6 | 2.68 | 163.19 | 30.66 |
| Gal(5)@O4 | **S244@N** | 2.89 | 151.52 | 20.91 |
| Fuc(4)@O4 | **N208@ND2** | 2.88 | 154.84 | 20.19 |
| **R242@O** | Glc(6)@O3 | 2.77 | 153.64 | 17.62 |
| **N208@OD1** | Fuc(4)@O4 | 2.77 | 151.71 | 17.45 |
| Fuc(1)@O4 | **T246@N** | 2.89 | 146.15 | 11.32 |
| **S244@OG** | GlcNAc(3)@O6 | 2.83 | 157.29 | 10.82 |
| Fuc(1)@H4O | **T246@N** | 2.92 | 151.27 | 10.35 |
| **Iso2** | | | | |
| Fuc(1)@O4 | **T210@OG1** | 2.79 | 158.18 | 84.95 |
| **G158@O** | Fuc(1)@O3 | 2.77 | 161.85 | 87.33 |
| **C156@O** | Fuc(1)@O4 | 2.77 | 149.24 | 65.40 |
| **Q202@OE1** | GlcNAc(3)@O6 | 2.77 | 159.78 | 62.73 |
| **Q202@OE1** | Gal(5)@O4 | 2.72 | 158.48 | 61.54 |
| **N161@O** | Fuc(1)@O2 | 2.79 | 159.40 | 39.94 |
| Gal(5)@O4 | **A208@N** | 2.89 | 150.10 | 33.95 |
| **H205@O** | Glc(6)@O2 | 2.78 | 155.38 | 17.46 |
| **Iso3** | | | | |
| **G191@O** | Fuc(1)@O3 | 2.78 | 163.17 | 87.77 |
| Fuc(1)@O4 | **T246@OG1** | 2.80 | 162.72 | 83.21 |
| **N194@O** | Fuc(1)@O2 | 2.76 | 160.26 | 71.98 |
| **C189@O** | Fuc(1)@O4 | 2.79 | 149.62 | 62.36 |
| **S244@OG** | Gal(5)@O4 | 2.81 | 157.11 | 56.87 |
| GlcNAc(3)@O6 | **S244@OG** | 2.80 | 159.74 | 55.71 |
| **D233@OD1** | GlcNAc(3)@O6 | 2.68 | 164.05 | 40.36 |
| **D233@OD2** | GlcNAc(3)@O6 | 2.68 | 164.20 | 38.32 |
| Gal(5)@O4 | **S244@N** | 2.90 | 153.13 | 28.54 |
| Fuc(4)@O4 | **N208@ND2** | 2.88 | 158.01 | 25.54 |
| Gal(5)@H40 | **S244@N** | 2.80 | 143.37 | 17.57 |
| **R242@O** | Glc(6)@O3 | 2.78 | 153.43 | 16.05 |
| Fuc(1)@O5 | **Q207@NE2** | 2.89 | 144.98 | 12.02 |
| **Iso4** | | | | |
| Fuc(1)@O4 | **T249@OG1** | 2.80 | 162.99 | 85.67 |
| **G192@O** | Fuc(1)@O3 | 2.78 | 162.17 | 83.99 |
| **N195@O** | Fuc(1)@O2 | 2.76 | 160.61 | 78.99 |
| GlcNAc(3)@O6 | **S247@OG** | 2.79 | 159.71 | 57.70 |
| **C190@O** | Fuc(1)@O4 | 2.80 | 147.99 | 55.81 |
| **S247@OG** | Gal(5)@O4 | 2.82 | 154.31 | 52.95 |
| Gal(5)@O4 | **S247@N** | 2.89 | 158.81 | 46.17 |
| **D236@OD2** | GlcNAc(3)@O6 | 2.68 | 163.41 | 42.65 |
| **D236@OD1** | GlcNAc(3)@O6 | 2.68 | 163.43 | 41.26 |
| Gal(5)@H40 | **S247@N** | 2.79 | 144.40 | 35.28 |
| Fuc(1)@O5 | **Q210@NE2** | 2.87 | 145.47 | 21.13 |
| Fuc(4)@O4 | **N211@ND2** | 2.88 | 157.24 | 18.63 |
| **E193@OE1** | ROH@O1 | 2.69 | 161.88 | 15.68 |
| GlcNAc(3)@O5 | **S247@OG** | 2.84 | 152.14 | 14.14 |
| GlcNAc(3)@H60 | **S247@OG** | 2.87 | 146.41 | 13.97 |
| **E193@OE2** | ROH@O1 | 2.70 | 161.46 | 13.30 |
| Glc(6)@O2 | **Q246@NE2** | 2.88 | 156.82 | 11.72 |
| **D193@OE2** | Glc(6)@O2 | 2.72 | 161.23 | 10.30 |

**Table S4:** The binding free energies of Iso1 in acidic condition along with their different energy components from the MM/GBSA scheme in kcal/mol. Standard deviation is given in the parenthesis.

| **Iso1 (Acidic)** | **ΔE_vdW_** | **ΔE_elec_** | **ΔG_pol_** | **ΔG_np_** | **ΔE_MM_^a^** | **ΔG_solv_^b^** | **ΔG_Total_^c^** |
| --- | --- | --- | --- | --- | --- | --- | --- |
| **Run1** | -38.53 | -68.42 | 79.40 | -5.70 | -106.95 | 73.71 | -33.25 |
| **Run2** | -40.53 | -71.60 | 83.03 | -6.06 | -112.13 | 76.98 | -35.15 |
| **Average** | -39.53 (1.00) | -70.01 (1.59) | 81.22 (1.82) | -5.88 (0.18) | -109.54 (2.59) | 75.35 (1.64) | **-34.20 (0.95)** |
| **a: ΔE_vdW +_ ΔE_elec_**  **b: ΔG_pol +_ ΔG_np_**  **c: ΔE_vdW +_ ΔE_elec +_ ΔG_pol +_ ΔG_np_** | | | | | | | |


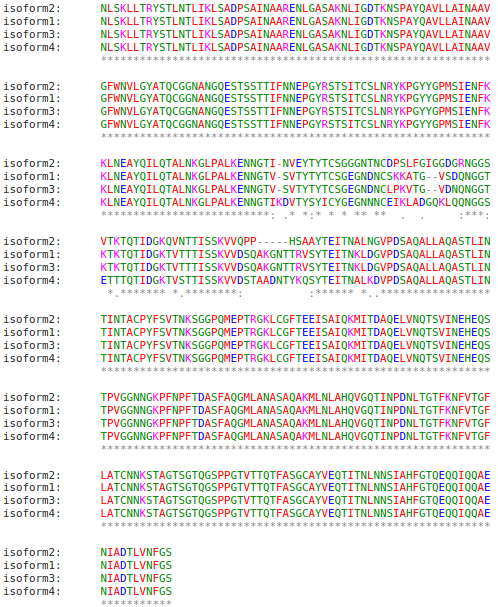


**Figure S1:** Multiple sequence alignment for all four isoforms of BabA. MSA was done using the Clustal Omega webserver^1^.


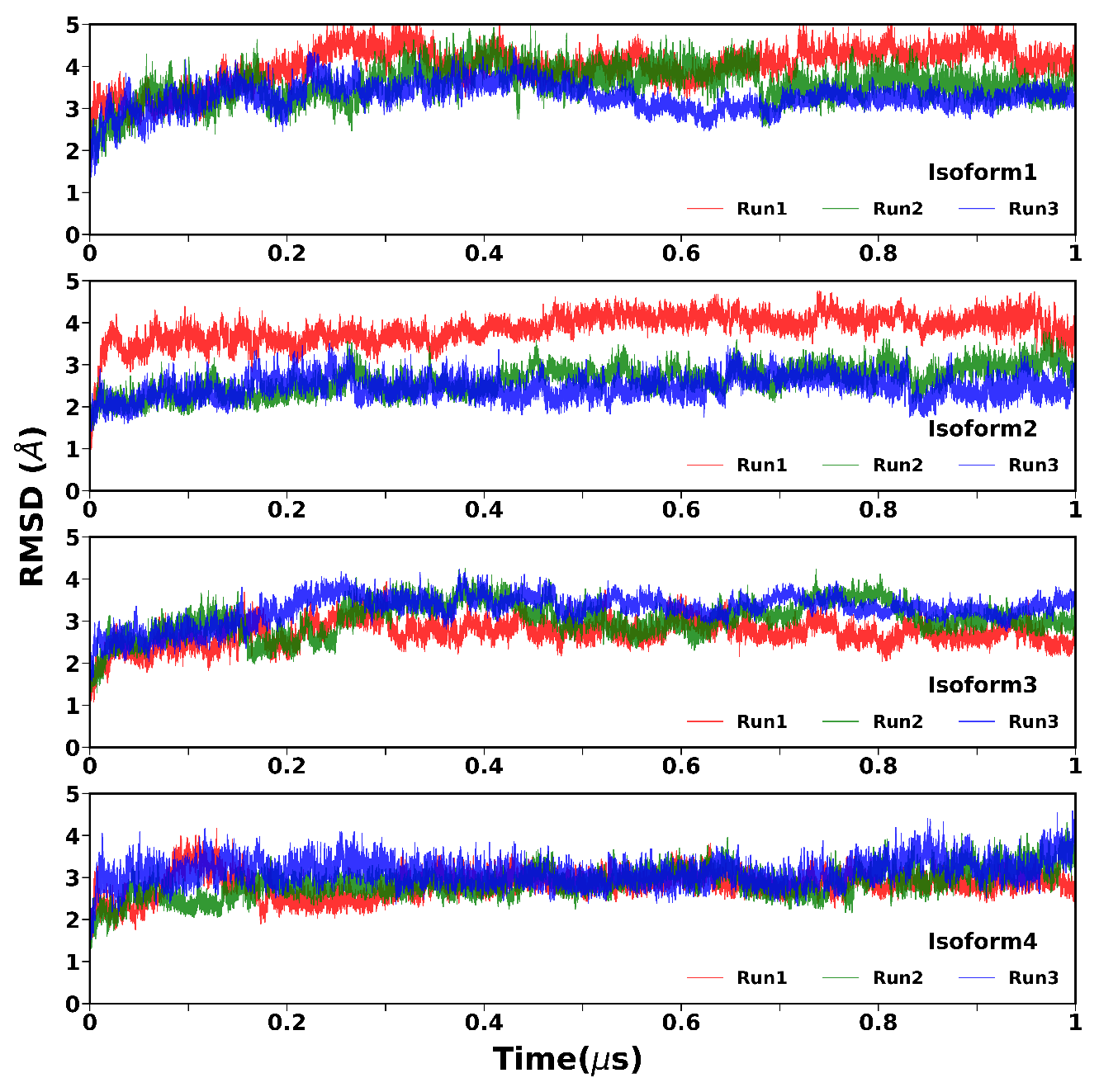


**Figure S2:** Time evolution of root mean squared deviations (RMSD) of backbone atoms of Isoforms of BabA (in Apo form). For each case, three replica runs are shown.


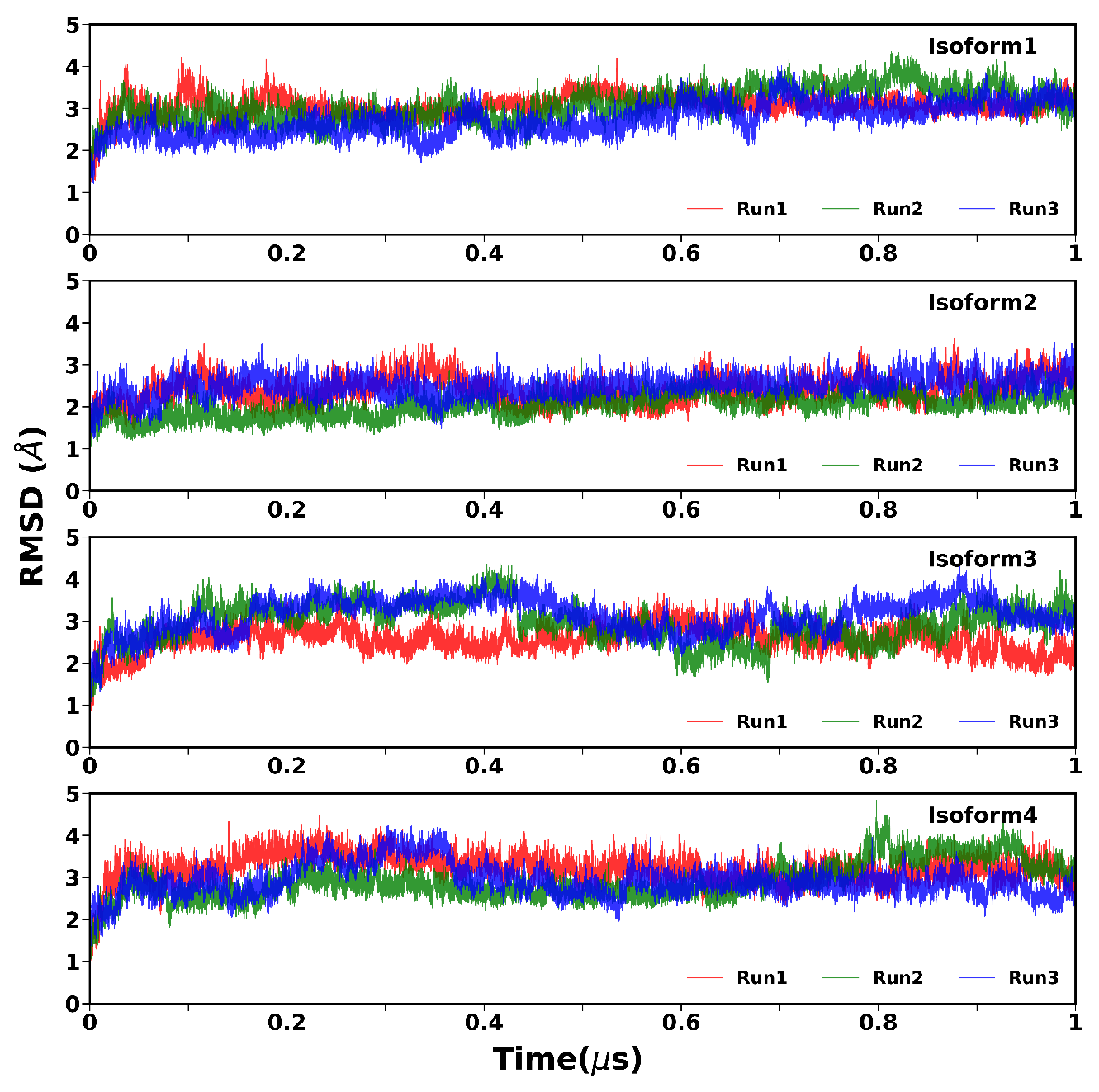


**Figure S3:** Time evolution of root mean squared deviations (RMSD) of backbone atoms of Isoforms of BabA (in Complex form). For each case, three replica runs are shown.


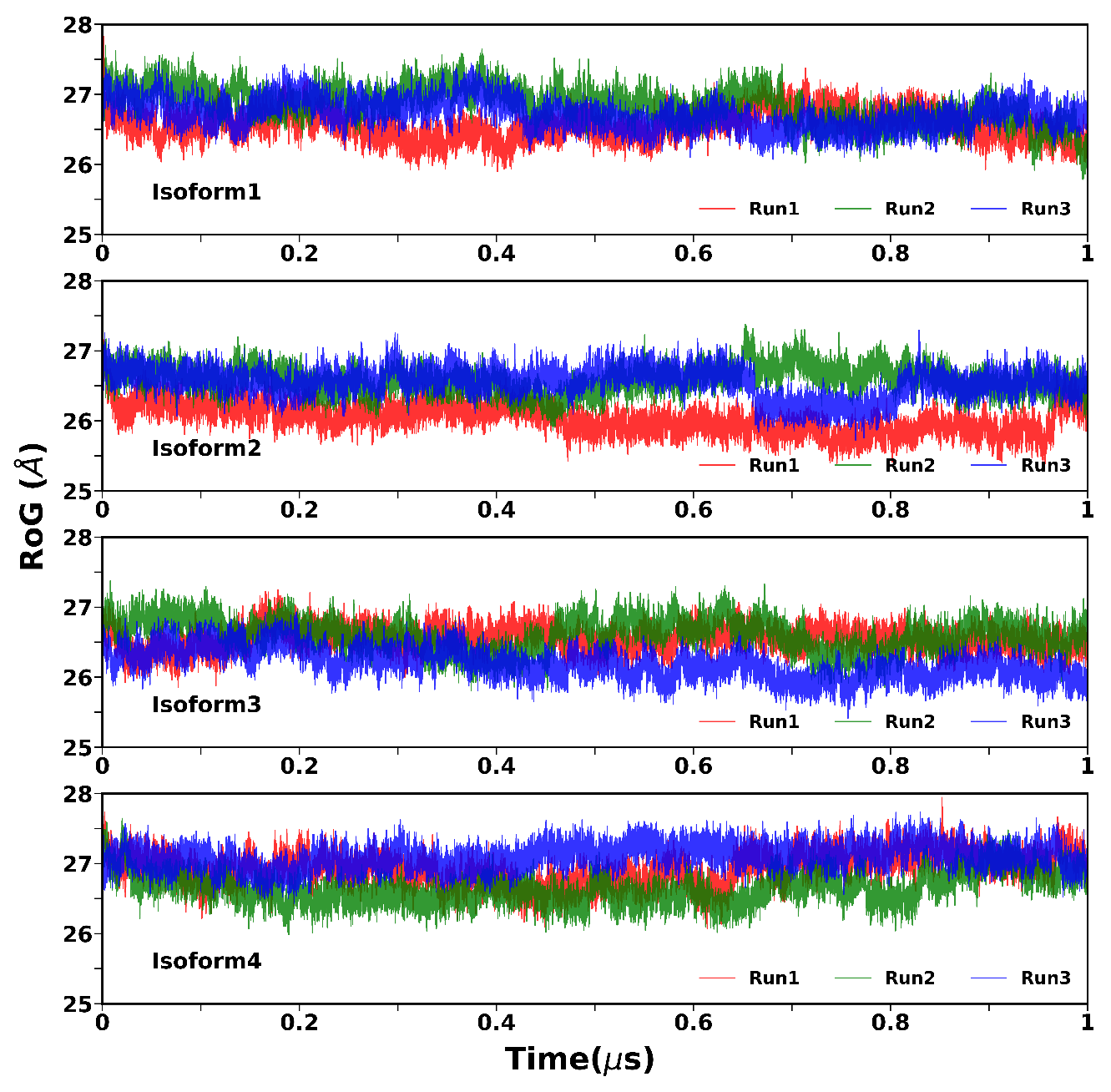


**Figure S4:** Time evolution of radius of gyration (RoG) of backbone atoms of Isoforms of BabA (in Apo form). For each case, three replica runs are shown.


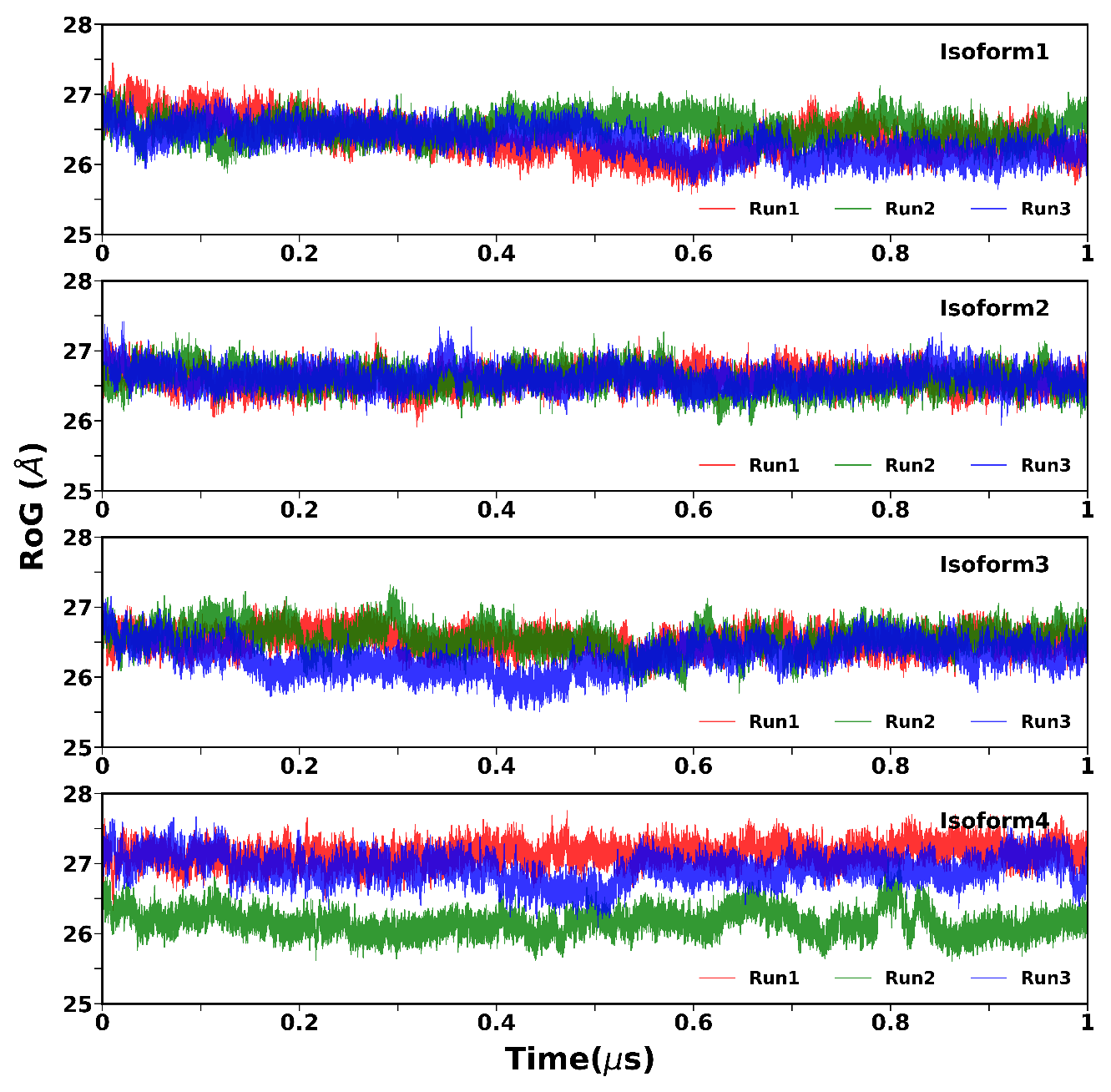


**Figure S5:** Time evolution of radius of gyration (RoG) of backbone atoms of Isoforms of BabA (in Complex form). For each case, three replica runs are shown.


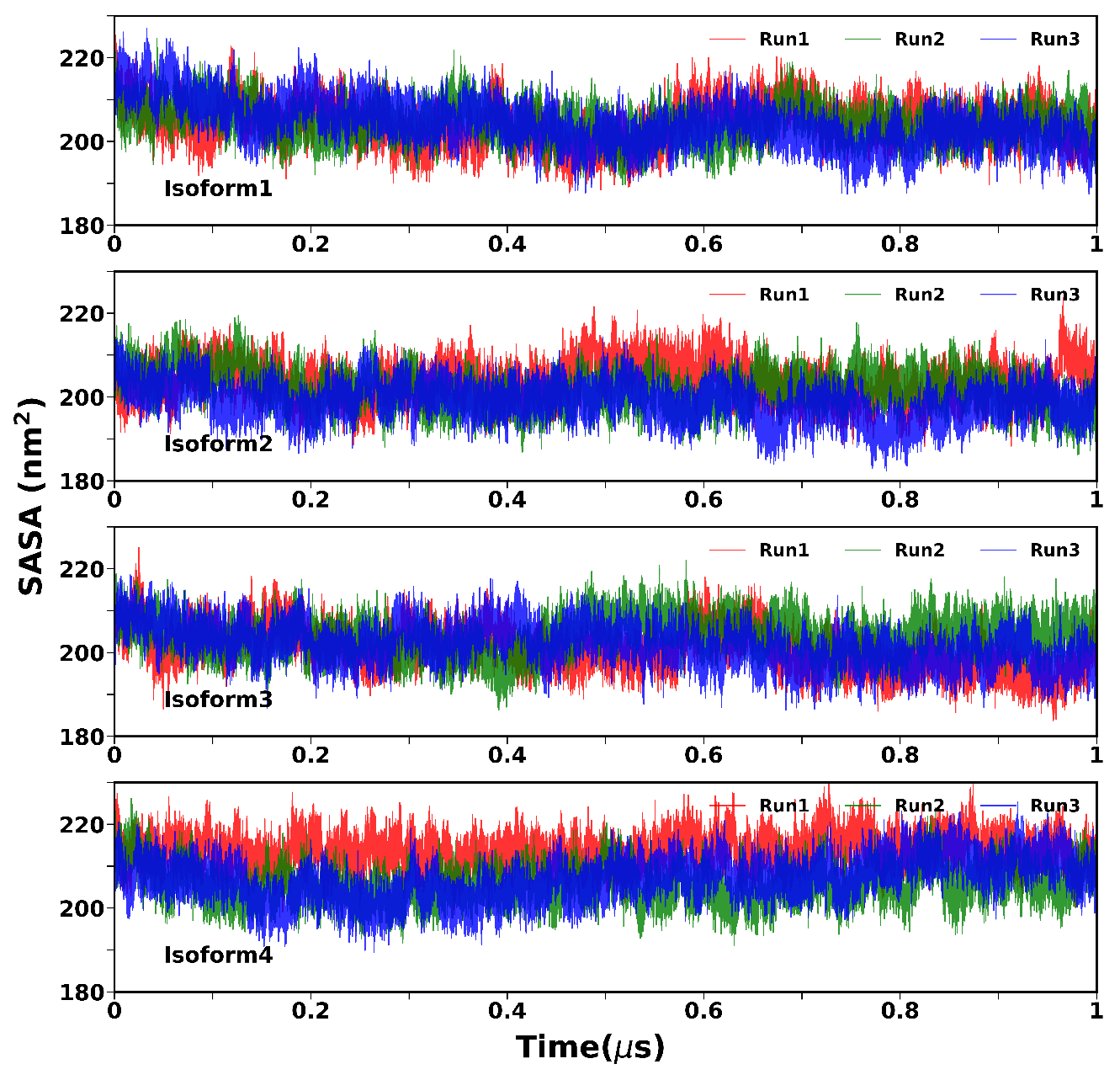


**Figure S6:** Time evolution of solvent accessible surface area (SASA) of backbone atoms of Isoforms of BabA (in Apo form). For each case, three replica runs are shown.


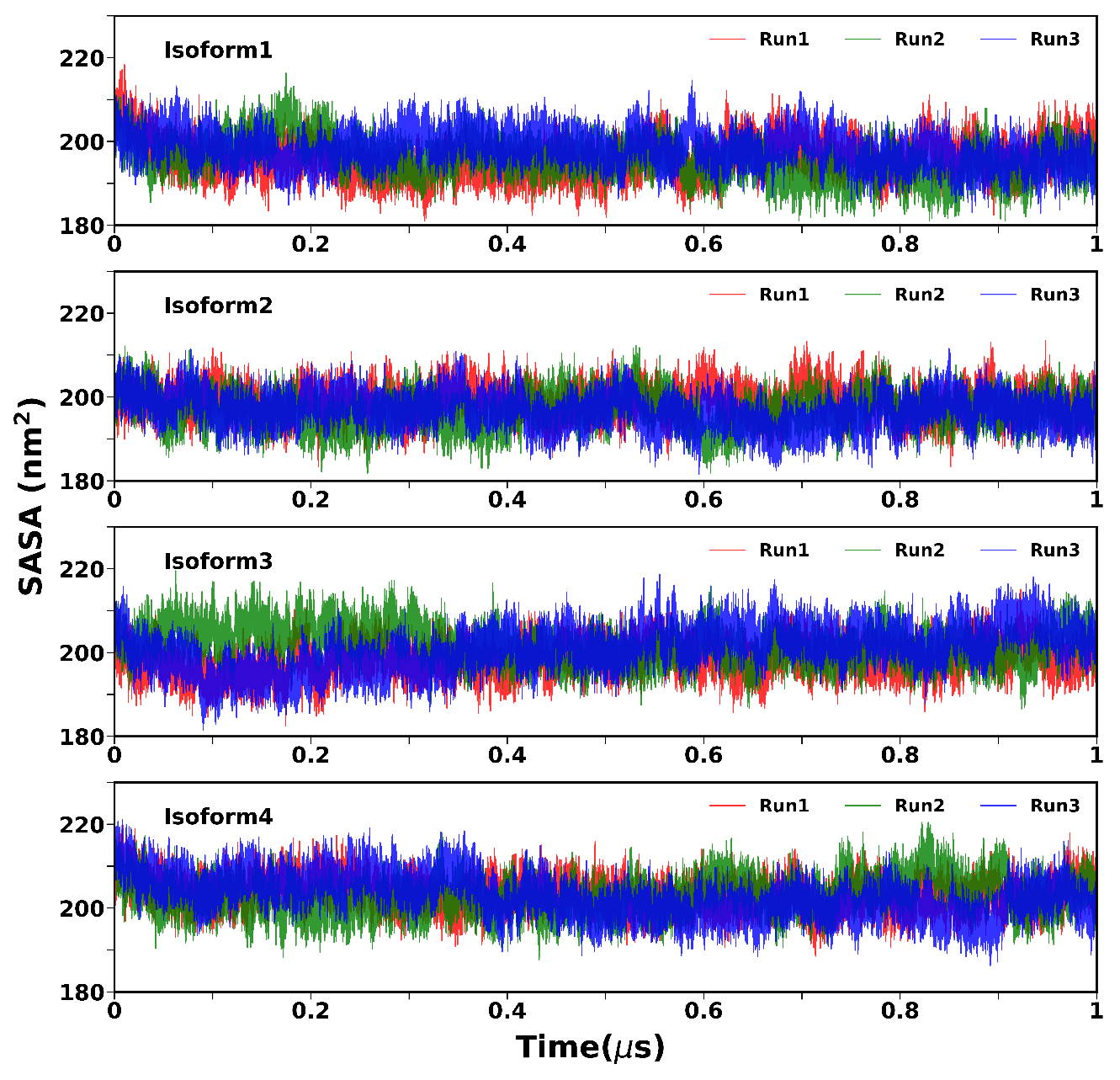


**Figure S7:** Time evolution of solvent accessible surface area (SASA) of backbone atoms of Isoforms of BabA (in Complex form). For each case, three replica runs are shown.


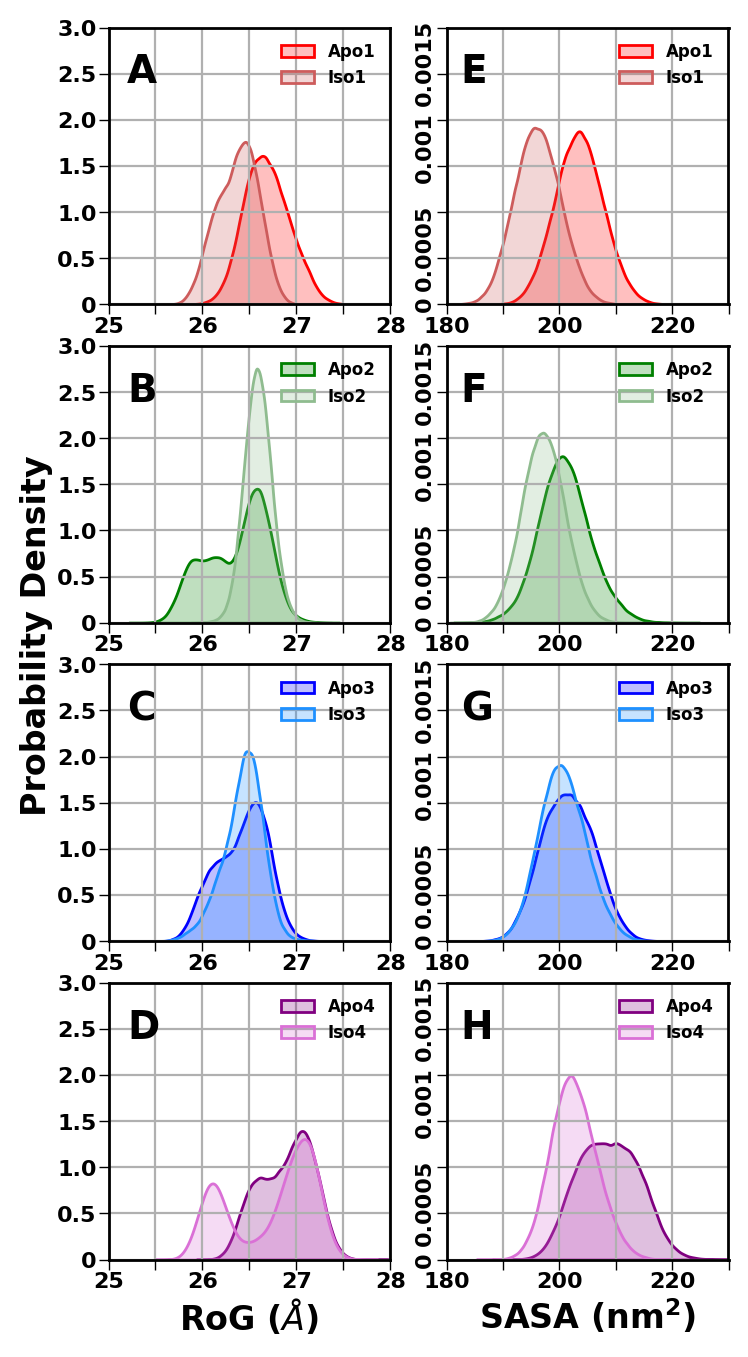


**Figure S8:** Distribution of radius of gyration (RoG) for Apo and Complex form A) Iso1, B) Iso2, C) Iso3 and D) Iso4. Distribution of solvent accessible surface area (SASA) for Apo and Complex form E) Iso1, F) Iso2, G) Iso3, H) Iso4.


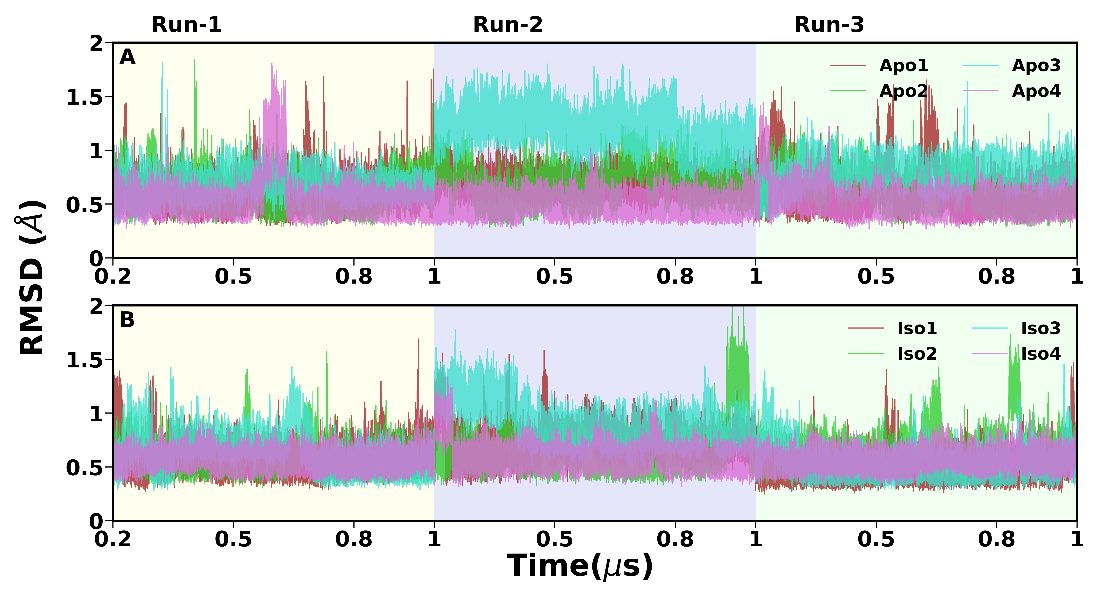


**Figure S9:** Time evolution of root mean square deviation (RMSD) of CL1 loop over three replicas run. For each run, initial 200 ns was not shown. A) Apo simulation, B) Complex simulation.


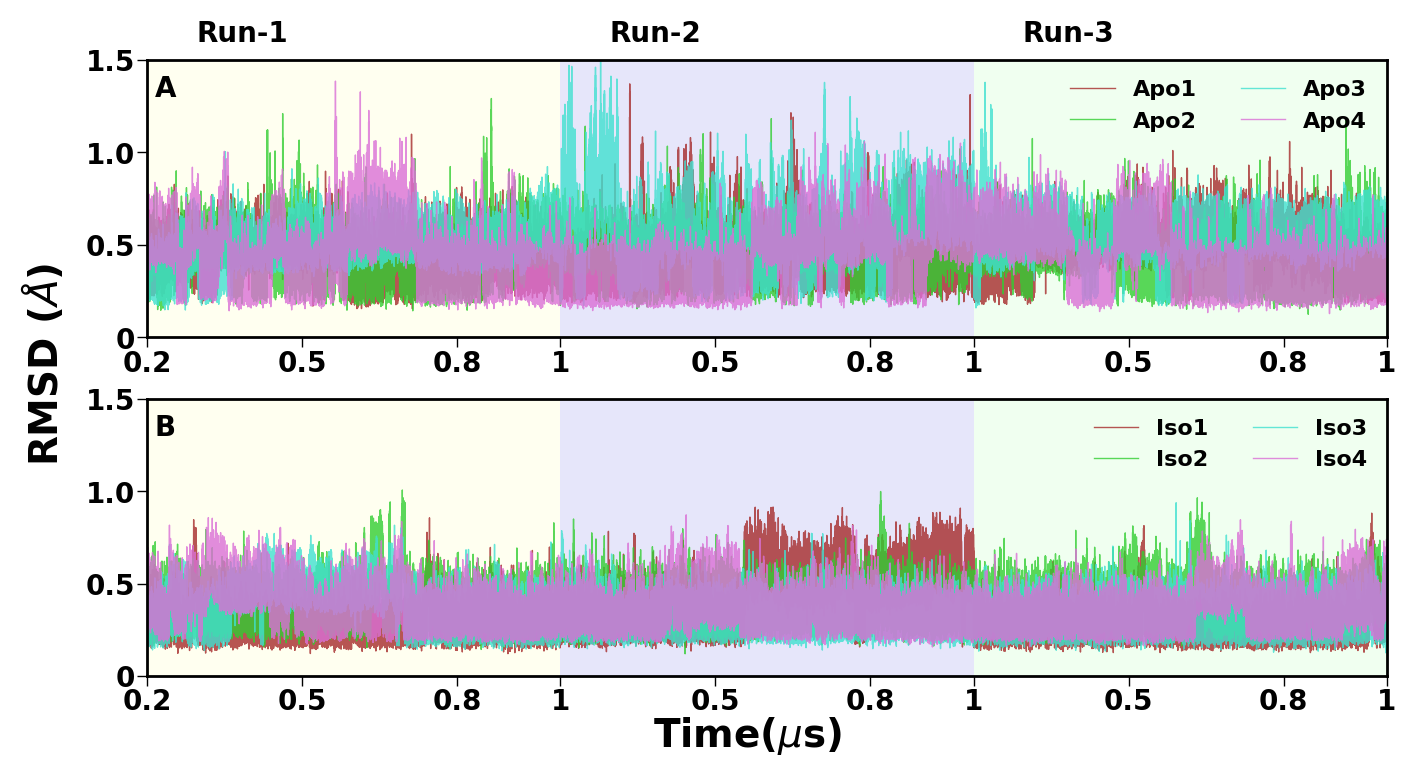


**Figure S10:** Time evolution of root mean square deviation (RMSD) of CL2 loop over three replicas run. For each run, initial 200 ns was not shown. A) Apo simulation, B) Complex simulation.


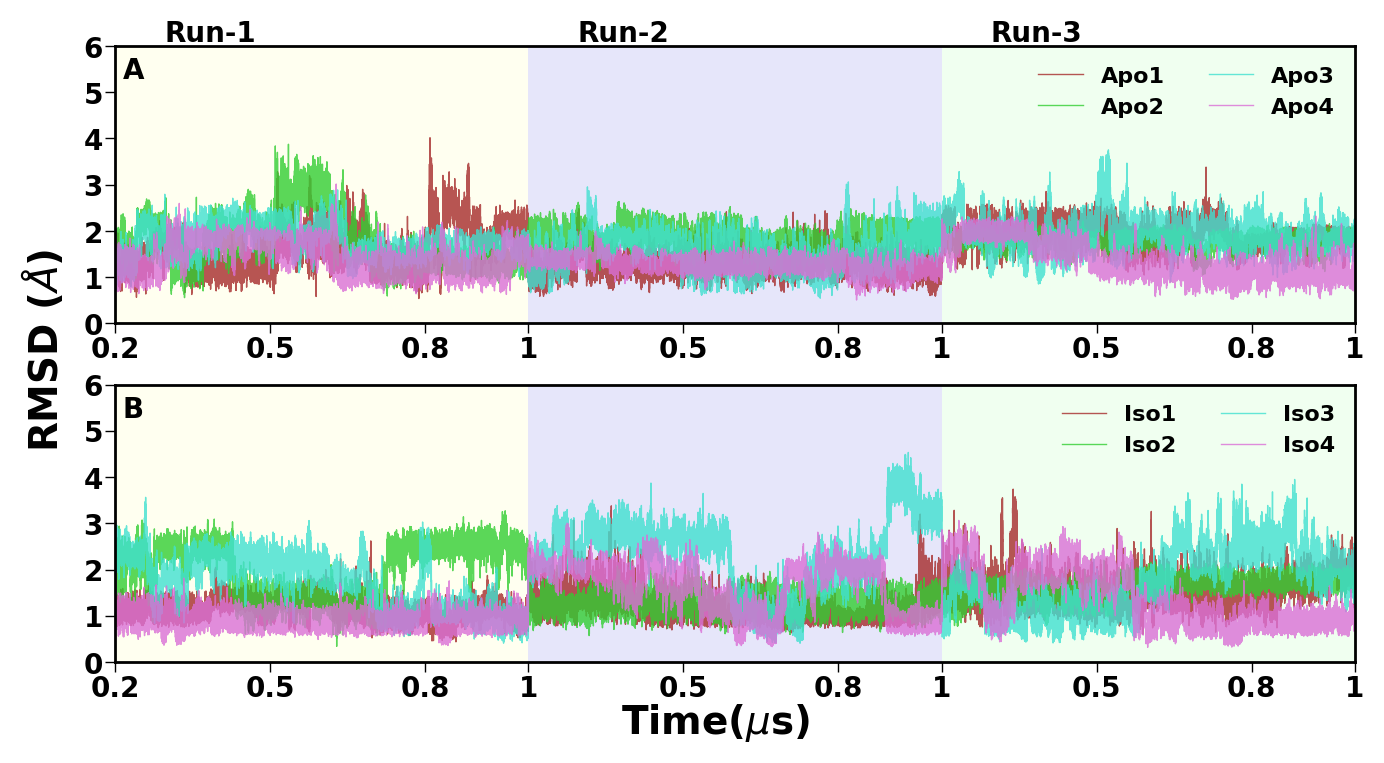


**Figure S11:** Time evolution of root mean square deviation (RMSD) of CL3 loop over three replicas run. For each run, initial 200 ns was not shown. A) Apo simulation, B) Complex simulation.


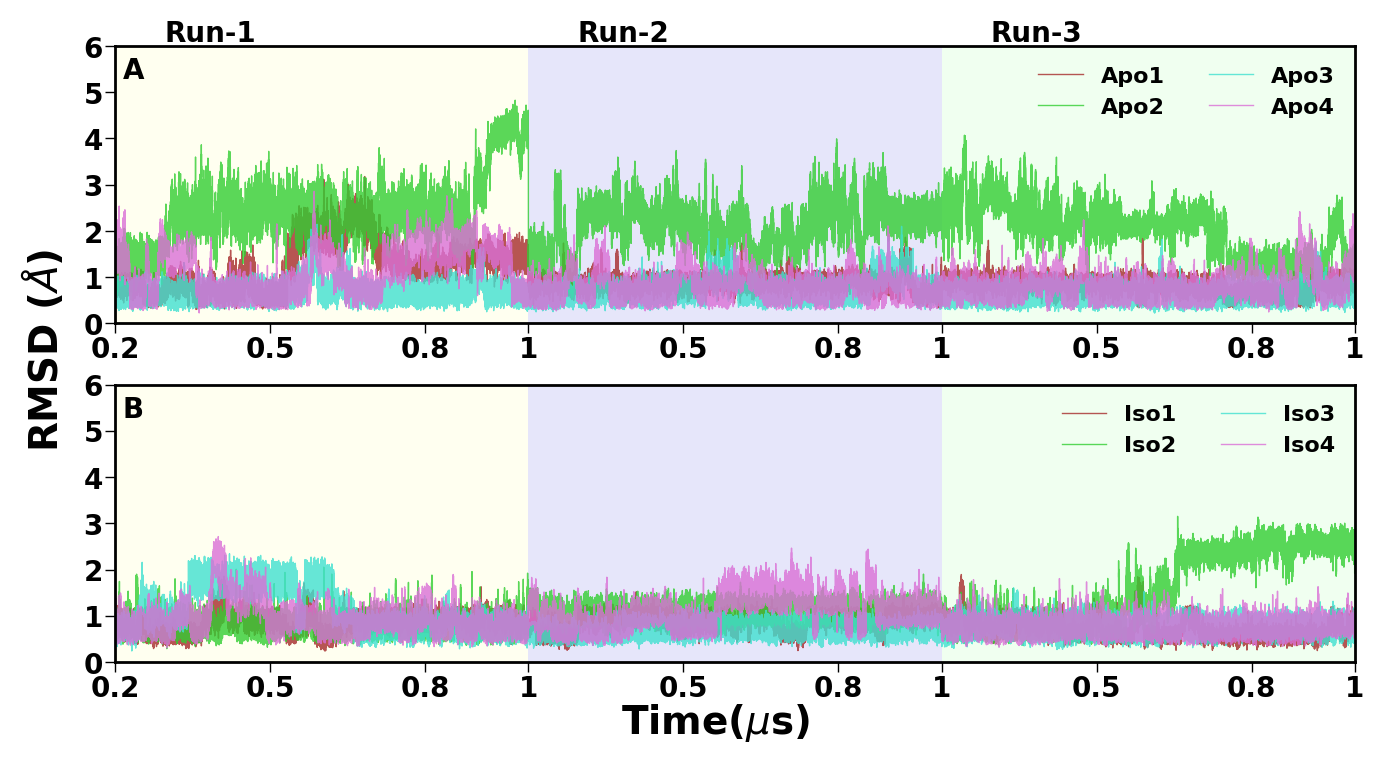


**Figure S12:** Time evolution of root mean square deviation (RMSD) of DL1 loop over three replicas run. For each run, initial 200 ns was not shown. A) Apo simulation, B) Complex simulation.


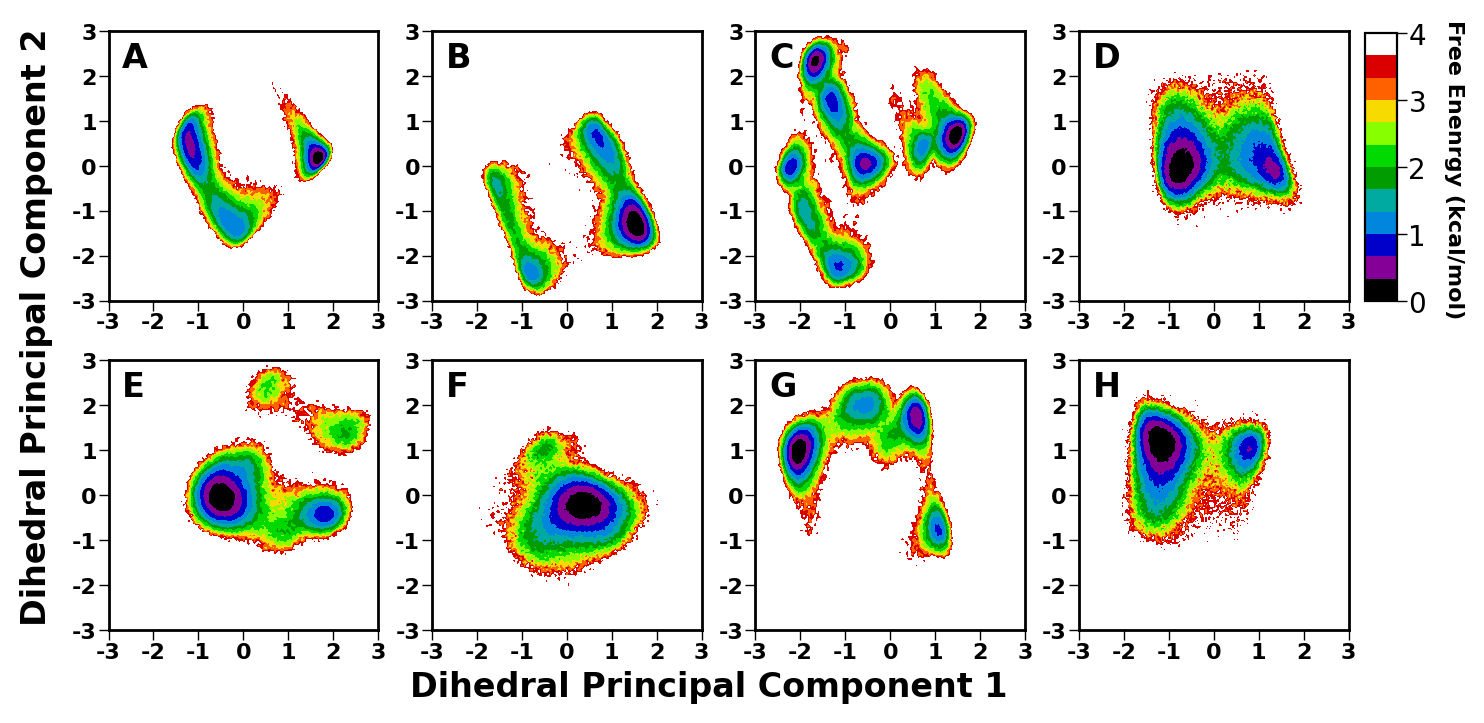


**Figure S13:** Free energy analysis of complete BabA (CL1) with respect to dihedral principal (dPCA) components, where dPC1 and dPC2 represents first and second principal components of motion. First row represents the Apo cases, A) Iso1, B) Iso2, C) Iso3, D) Iso4 whereas second row represents the complex simulations, E) Iso1, F) Iso2, G) Iso3, H) Iso4.


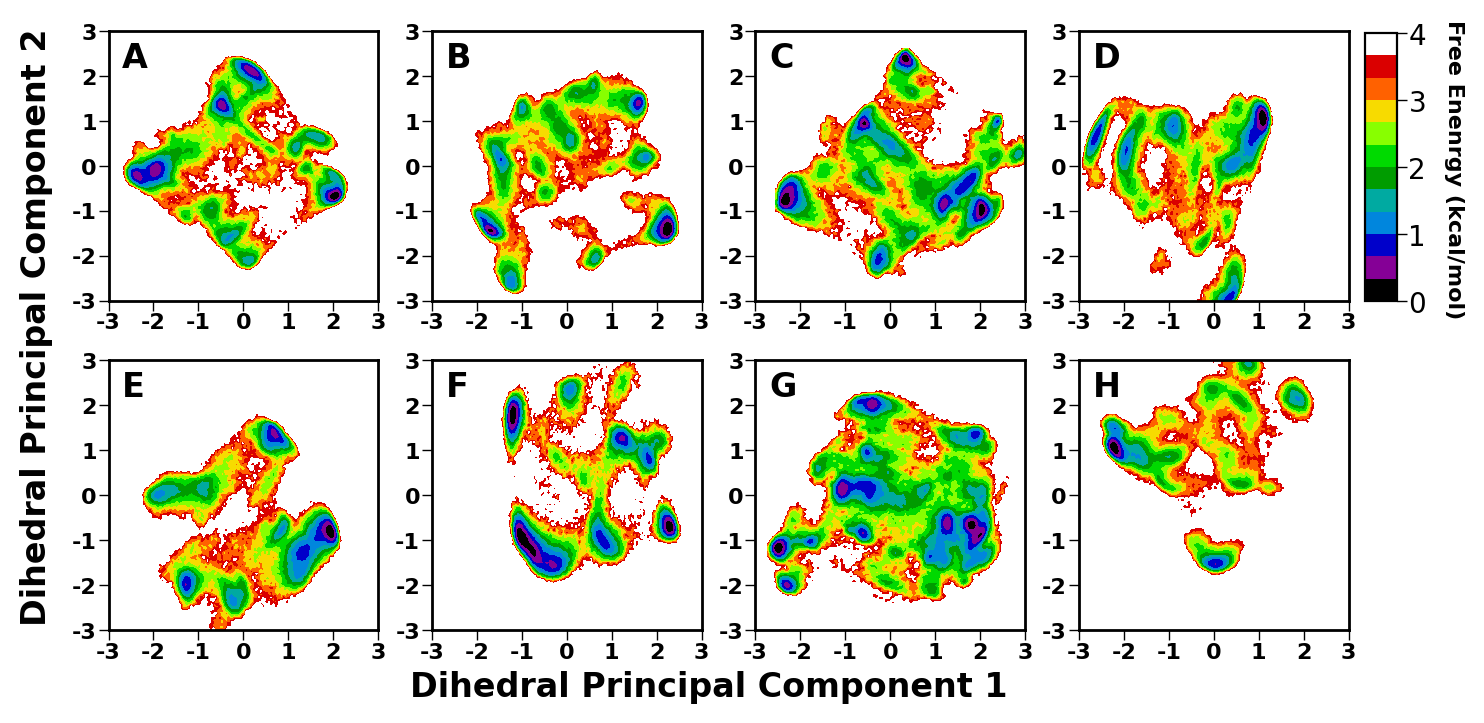


**Figure S14:** Free energy analysis of complete BabA (CL3) with respect to dihedral principal (dPCA) components, where dPC1 and dPC2 represents first and second principal components of motion. First row represents the Apo cases, A) Iso1, B) Iso2, C) Iso3, D) Iso4 whereas second row represents the complex simulations, E) Iso1, F) Iso2, G) Iso3, H) Iso4.


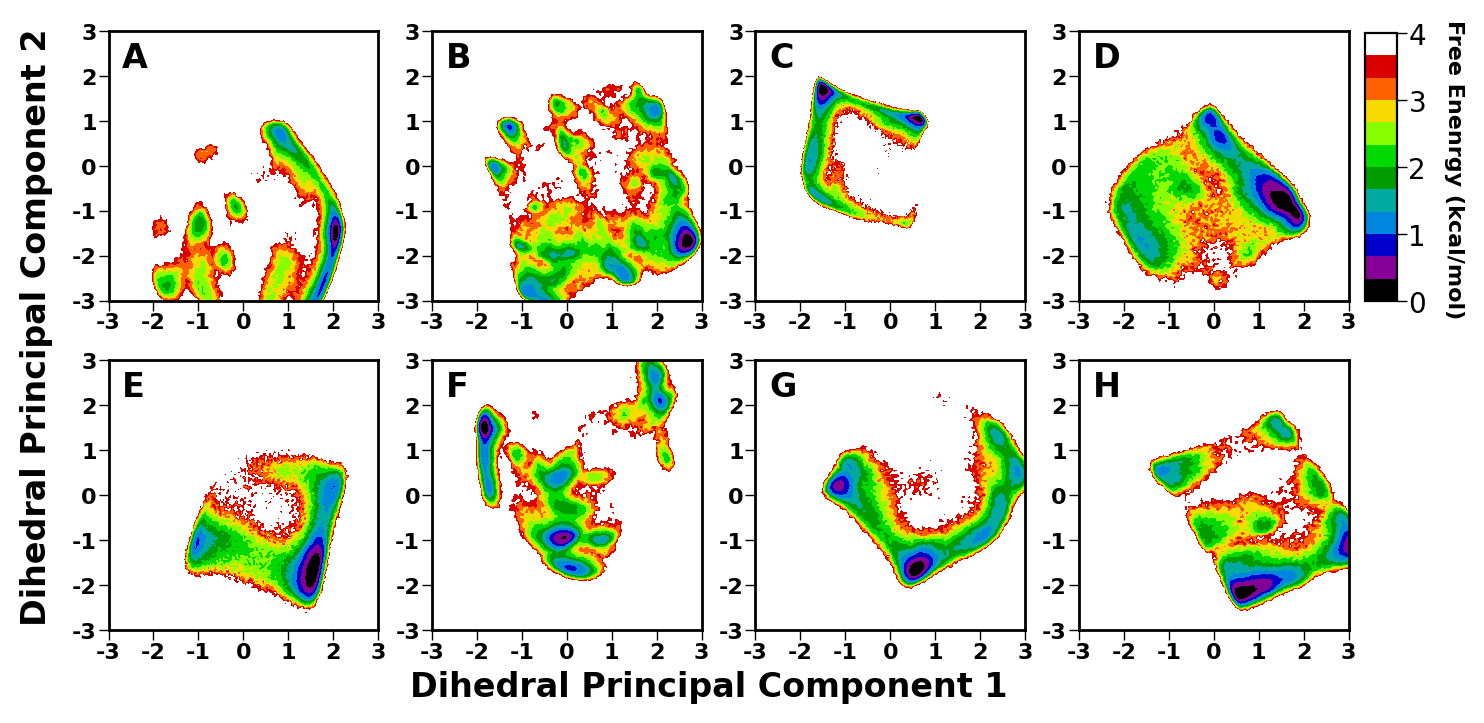


**Figure S15:** Free energy analysis of complete BabA (DL1) with respect to dihedral principal (dPCA) components, where dPC1 and dPC2 represents first and second principal components of motion. First row represents the Apo cases, A) Iso1, B) Iso2, C) Iso3, D) Iso4 whereas second row represents the complex simulations, E) Iso1, F) Iso2, G) Iso3, H) Iso4.


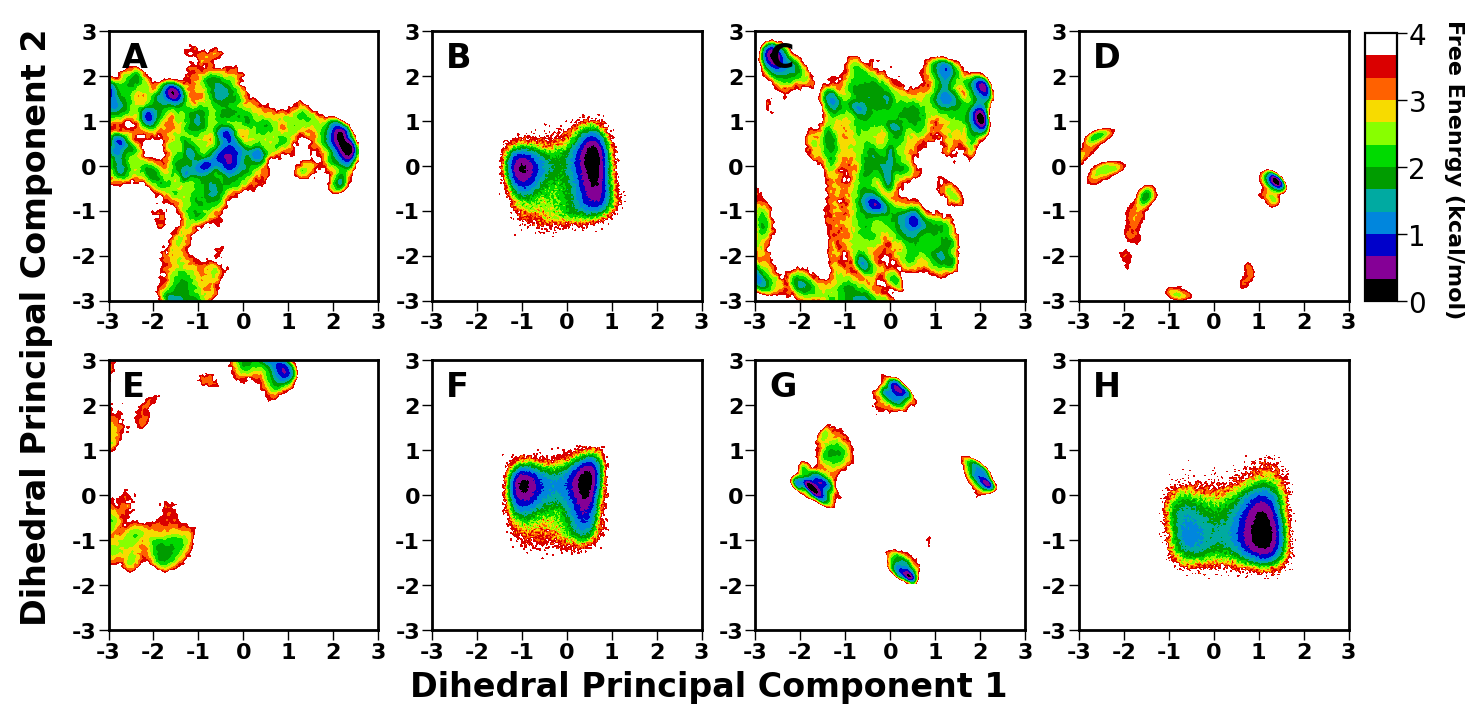


**Figure S16:** Free energy analysis of complete BabA (DL2) with respect to dihedral principal (dPCA) components, where dPC1 and dPC2 represents first and second principal components of motion. First row represents the Apo cases, A) Iso1, B) Iso2, C) Iso3, D) Iso4 whereas second row represents the complex simulations, E) Iso1, F) Iso2, G) Iso3, H) Iso4.


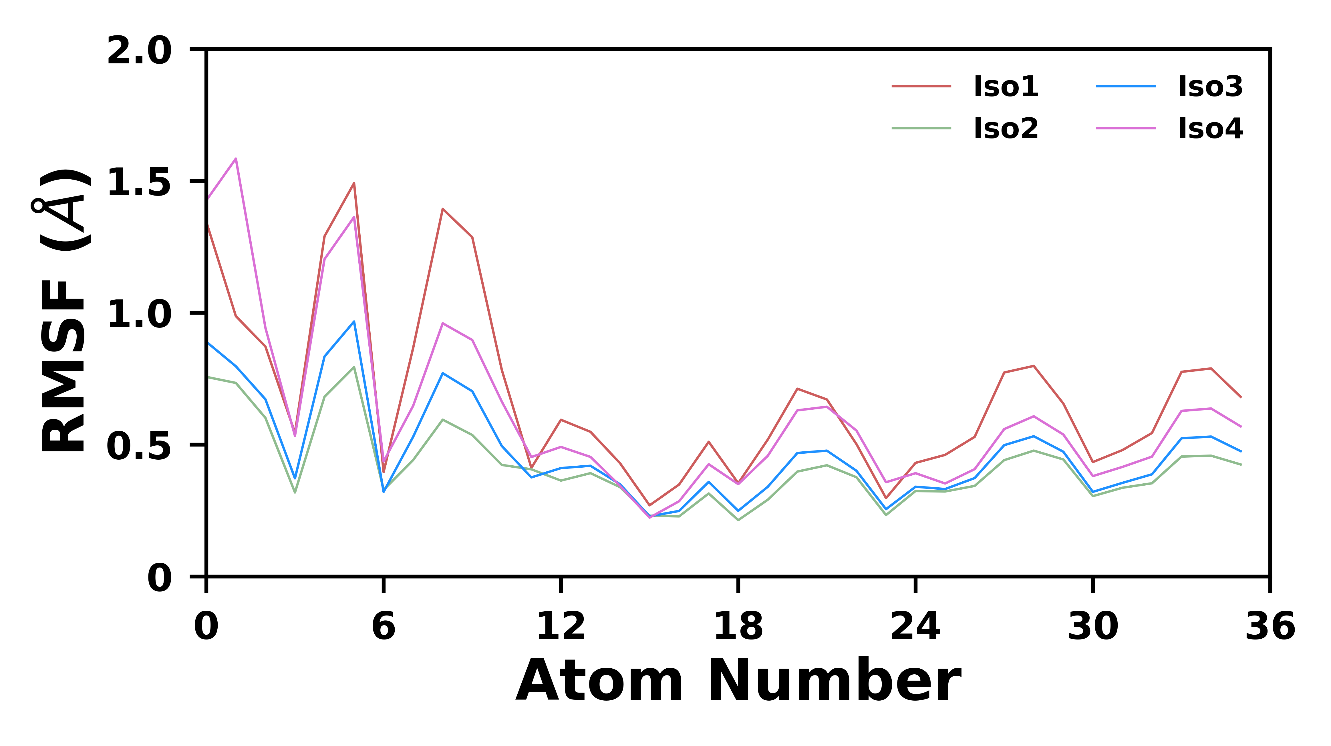


**Figure S17:** The root mean squared fluctuation (RMSF) of Le^b^ in complex with four Isoforms of BabA.


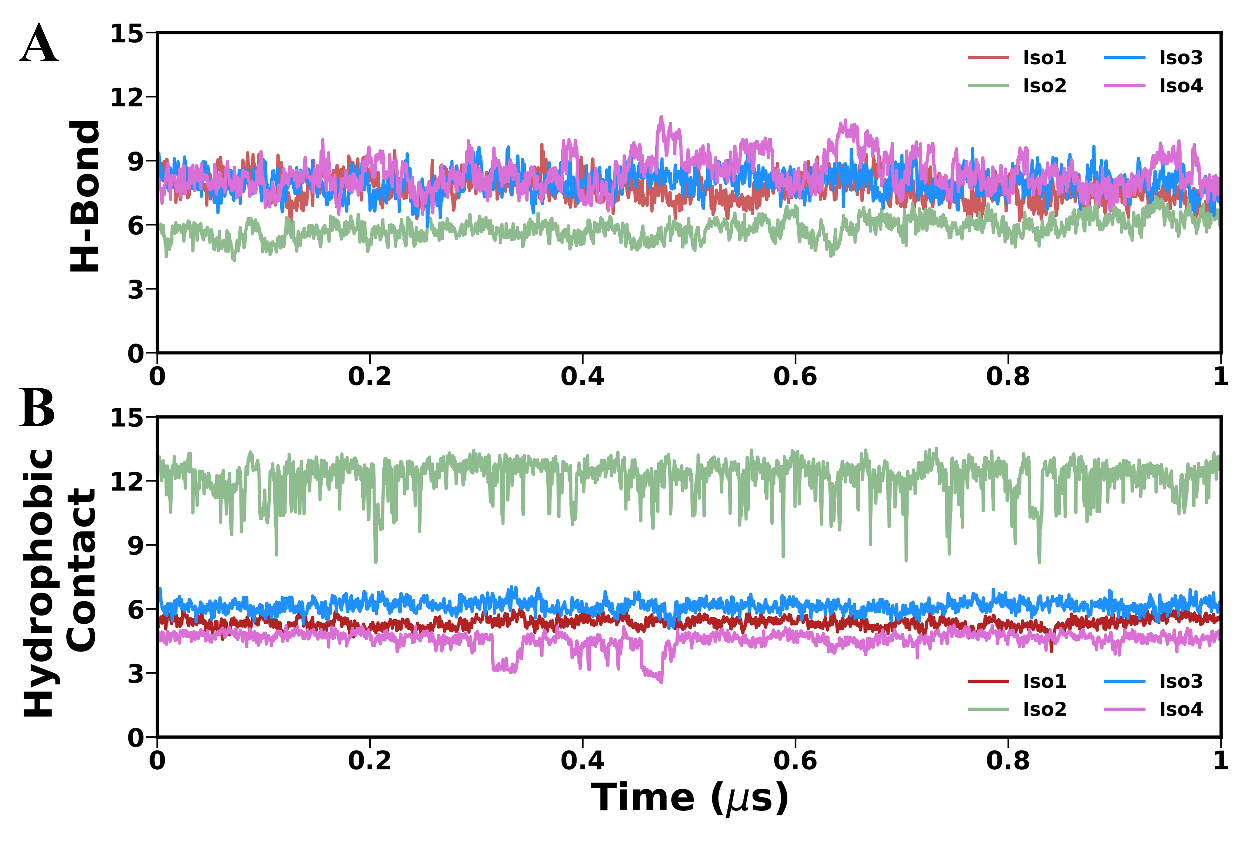


**Figure S18:** A) Time evolution of hydrogen bonds (upper column) and B) Hydrophobic contacts (lower column) between BabA and Le^b^. Data are represented by averaging the three replica runs.


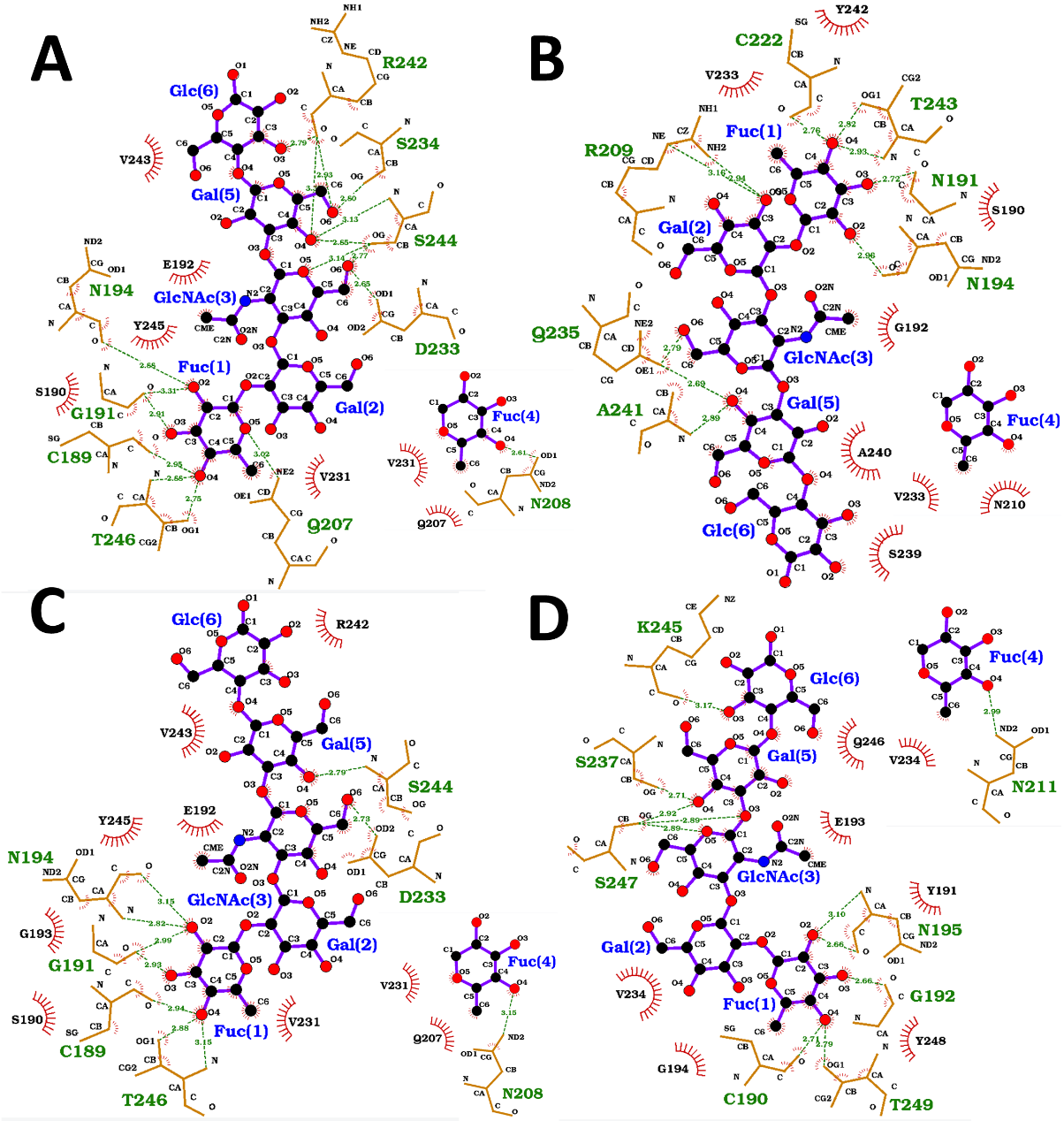


**Figure 19:** 2D representation of hydrogen bonds and hydrophobic contact using Ligplot^42^ for A) Iso1, B) Iso2, C) Iso3, D) Iso4 complexes.


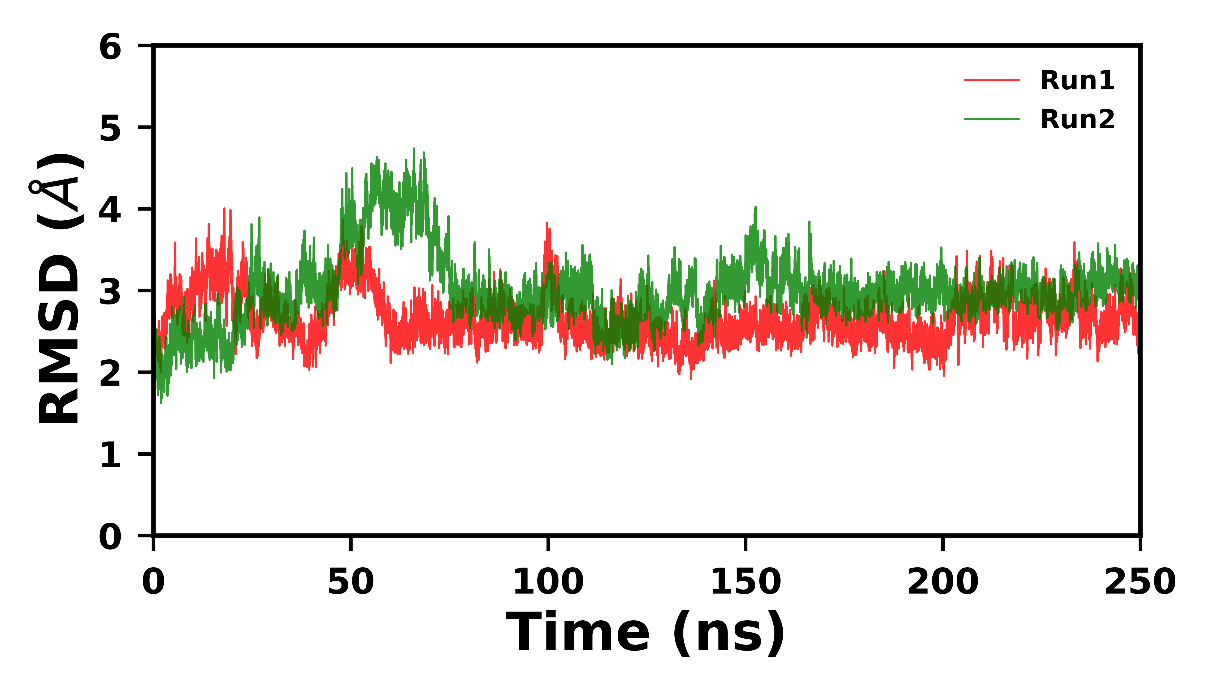


**Figure S20:** Time evolution of root mean squared deviation (RMSD) of BabA (Iso1) in acidic condition.


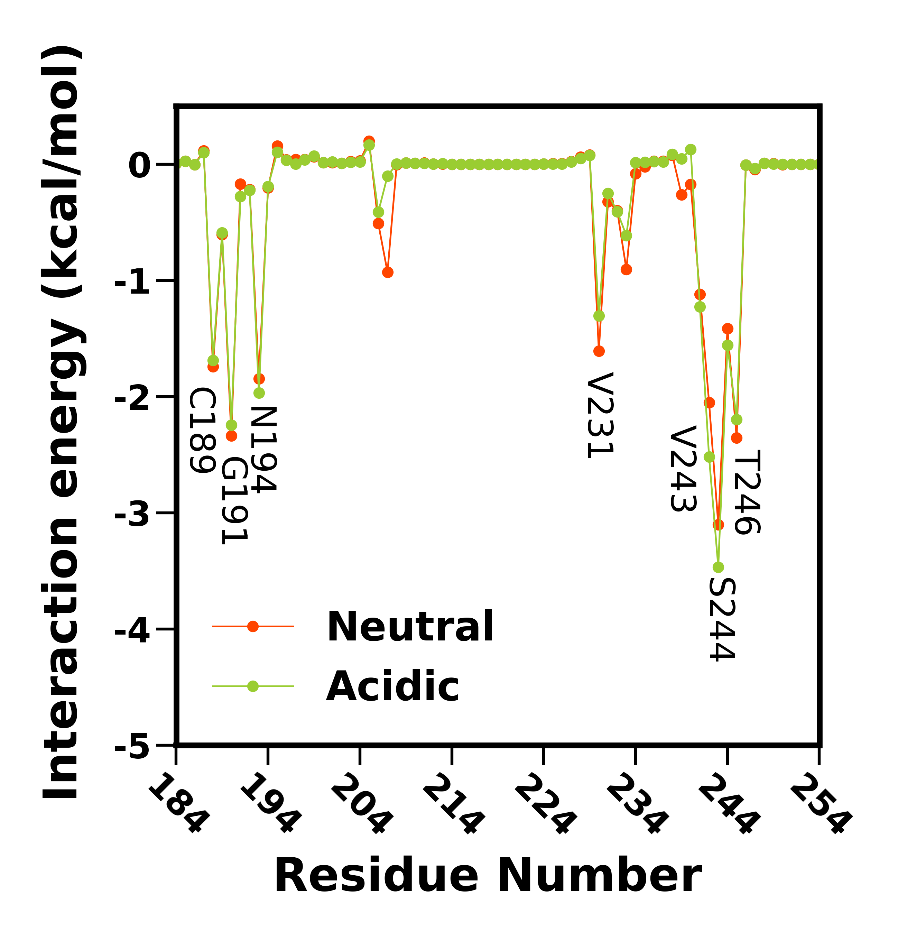


**Figure S21:** Per residue-wise contribution to the binding free energy of BabA and Le^b^ complexes in neutral and acidic condition.
